# HTLV-1 bZIP Factor Enhances T-Cell Proliferation by Impeding the Suppressive Signaling of Co-inhibitory Receptors

**DOI:** 10.1371/journal.ppat.1006120

**Published:** 2017-01-03

**Authors:** Haruka Kinosada, Jun-ichirou Yasunaga, Kazuya Shimura, Paola Miyazato, Chiho Onishi, Tomonori Iyoda, Kayo Inaba, Masao Matsuoka

**Affiliations:** 1 Laboratory of Virus Control, Institute for Virus Research, Kyoto University, Sakyo-ku, Kyoto, Japan; 2 Department of Mammalian Regulatory Network, Graduate School of Biostudies, Kyoto University, Sakyo-ku, Kyoto, Japan; 3 Laboratory of Immunology, Department of Animal Development and Physiology, Division of Systemic Life Science, Graduate School of Biostudies, Kyoto University, Sakyo-ku, Kyoto, Japan; 4 Department of Hematology, Rheumatology and Infectious Diseases, Kumamoto University School of Medicine, Kumamoto, Japan; University of Illinois at Chicago College of Medicine, UNITED STATES

## Abstract

Human T-cell leukemia virus type 1 (HTLV-1) causes adult T-cell leukemia-lymphoma (ATL) and inflammatory diseases. To enhance cell-to-cell transmission of HTLV-1, the virus increases the number of infected cells *in vivo*. HTLV-1 bZIP factor (HBZ) is constitutively expressed in HTLV-1 infected cells and ATL cells and promotes T-cell proliferation. However, the detailed mechanism by which it does so remains unknown. Here, we show that HBZ enhances the proliferation of expressing T cells after stimulation via the T-cell receptor. HBZ promotes this proliferation by influencing the expression and function of multiple co-inhibitory receptors. HBZ suppresses the expression of BTLA and LAIR-1 in HBZ expressing T cells and ATL cells. Expression of T cell immunoglobulin and ITIM domain (TIGIT) and Programmed cell death 1 (PD-1) was enhanced, but their suppressive effect on T-cell proliferation was functionally impaired. HBZ inhibits the co-localization of SHP-2 and PD-1 in T cells, thereby leading to impaired inhibition of T-cell proliferation and suppressed dephosphorylation of ZAP-70 and CD3ζ. HBZ does this by interacting with THEMIS, which associates with Grb2 and SHP-2. Thus, HBZ interacts with the SHP containing complex, impedes the suppressive signal from PD-1 and TIGIT, and enhances the proliferation of T cells. Although HBZ was present in both the nucleus and the cytoplasm of T cells, HBZ was localized largely in the nucleus by suppressed expression of THEMIS by shRNA. This indicates that THEMIS is responsible for cytoplasmic localization of HBZ in T cells. Since THEMIS is expressed only in T-lineage cells, HBZ mediated inhibition of the suppressive effects of co-inhibitory receptors accounts for how HTLV-1 induces proliferation only of T cells *in vivo*. This study reveals that HBZ targets co-inhibitory receptors to cause the proliferation of infected cells.

## Author Summary

Since HTLV-1 infects only through cell-to-cell transmission, increasing the number of infected cells is critical for transmission of HTLV-1. Proliferation of HTLV-1 infected cells is critical for development of leukemia and inflammatory diseases. In this study, we showed that HBZ promotes the proliferation of infected cells by targeting co-inhibitory receptors. Paradoxically, HBZ enhances the expression of the co-inhibitory receptors TIGIT and PD-1. We found that HBZ concurrently hampers the growth-inhibitory signal of TIGIT and PD-1, thereby leading to the enhanced proliferation of HTLV-1 infected cells *in vivo*. HBZ does this by interacting with THEMIS, which is expressed only in T cells. It is known that HTLV-1 infects different types of cells but increases only T cells. Functional impairment of co-inhibitory receptors by interaction of HBZ with THEMIS is a mechanism how HTLV-1 specifically induces proliferation of T cells.

## Introduction

Human T-cell leukemia virus type 1 (HTLV-1) belongs to the delta type retrovirus group, which also includes bovine leukemia virus and HTLV-2. HTLV-1 causes adult T-cell leukemia-lymphoma (ATL) and inflammatory diseases [1–4]. This virus induces clonal proliferation of infected cells to enhance its transmission, since HTLV-1 is transmitted primarily by cell-to-cell contact [5–7]. It has been reported that an increased number of infected cells is correlated with a higher rate of transmission by breast-feeding [8]. Thus, increased numbers of HTLV-1 infected cells are beneficial for the transmission of this virus.

HTLV-1 encodes two regulatory genes, *tax* and *rex*, and three accessory genes (*p12*, *p13*, and *p30*) in the plus strand of the provirus [2]. Another regulatory gene, the *HTLV-1 bZIP factor* (*HBZ*) gene, is transcribed as an anti-sense transcript [9, 10]. It has been reported that HTLV-1 infected cells show higher susceptibility to antigenic stimulation. One mechanism of this hypersensitivity is due to Tax. Tax expression under control of the long terminal repeat (LTR) results in enhanced responsiveness to stimulation through the T-cell receptor (TCR)/CD3 complex [11, 12]. However, Tax expression is often lost in ATL cells and HTLV-1 infected cells [13–18]. Therefore, it is likely that another mechanism also promotes proliferation of HTLV-1 infected cells–perhaps a mechanism involving HBZ. Indeed, HBZ has been reported to promote proliferation of T cells *in vivo* and *in vitro* [19, 20].

The TCR recognizes cognate antigenic peptides presented by major histocompatibility complex molecules on antigen-presenting cells, and transduces a signal that is modulated by co-stimulatory and co-inhibitory receptors on the T cell [21, 22]. It has been reported that ATL cells and HTLV-1 infected cells express the co-inhibitory receptors PD-1 and T cell immunoglobulin and ITIM domain (TIGIT) on their surfaces [23–25]. Binding of one of these receptors to its ligand sends a suppressive signal through the ITIM or ITSM motif in the cytoplasmic region of the receptor [21]. However, ATL cells and HTLV-1 infected cells proliferate regardless of the higher expression of PD-1 and TIGIT on their surfaces. Until now, it has not been known how the suppressive signal from these co-inhibitory receptors is impaired.

In this study, we found that HBZ promotes T-cell proliferation mediated by TCR signaling. As a mechanism, HBZ interferes with the suppressive function of some co-inhibitory receptors and inhibits the expression of others. Thus, impairment of co-inhibitory receptors is a newly discovered mechanism by which HTLV-1 promotes the proliferation of infected T cells.

## Results

### Proliferation of CD4^+^ T cells of HBZ transgenic mice is promoted upon TCR stimulation

**Fig 1:**
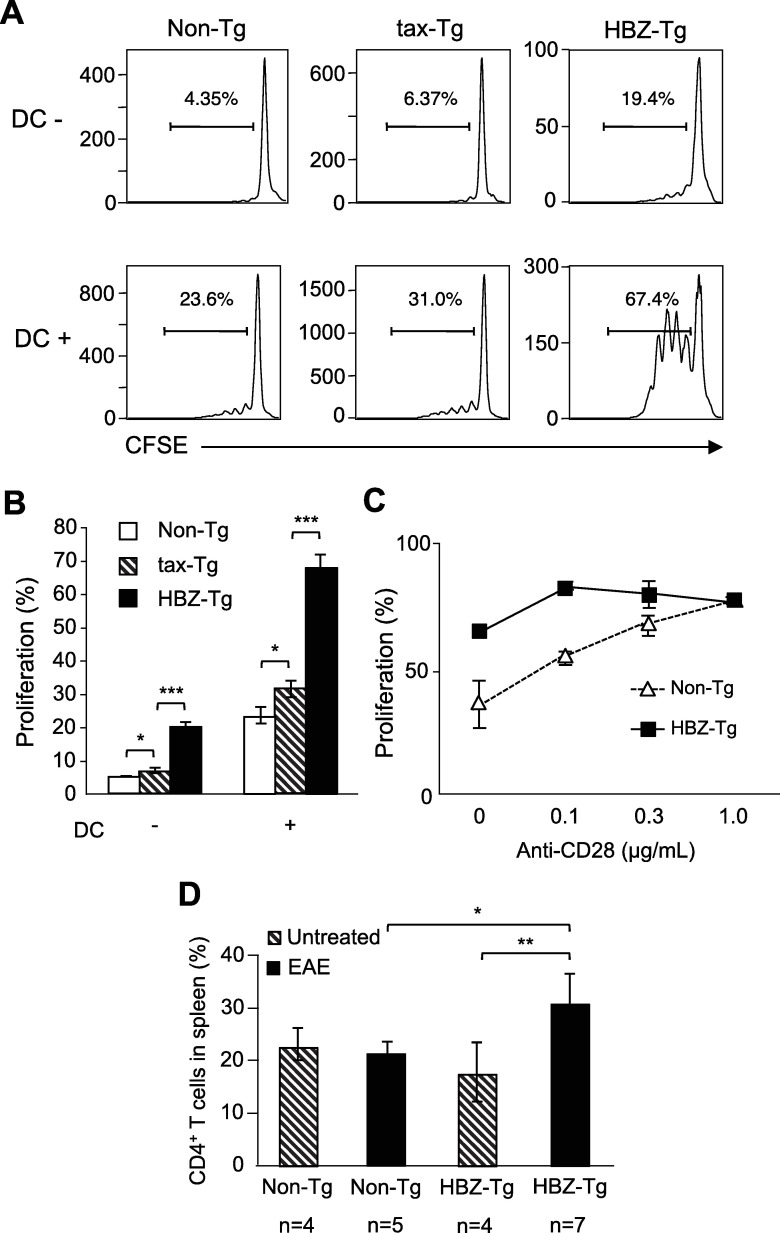
HBZ promotes proliferation of CD4^+^ T cells upon TCR stimulation. (A, B) Proliferation of CD4^+^ T cells isolated from non-Tg, tax-Tg and HBZ-Tg mice was analyzed by CFSE dilution. CD4^+^ T cells were stimulated with soluble anti-CD3 antibody (30 ng/mL) and cultured with or without dendritic cells for three days. (C) CD4^+^ T cells of non-Tg and HBZ-Tg mice were stimulated with immobilized anti-CD3 antibody (200 ng/mL) and soluble anti-CD28 antibody (0, 0.1, 0.3 and 1 μg/mL). CFSE dilution was analyzed by flow cytometry. (D) Experimental allergic encephalomyelitis (EAE) was induced in non-Tg and HBZ-Tg mice by immunization with MOG/CFA. The percentages of CD4^+^ T cells in spleen were measured in non-Tg and HBZ-Tg mice.

We have reported that *HBZ* promotes proliferation of a human T-cell line and *HBZ* knockdown inhibits proliferation of ATL cell lines [19]. Several mechanisms were identified for proliferation induced by HBZ [20, 26–31]. However, it remains unknown how HTLV-1 induces T-cell specific proliferation. We generated HBZ transgenic (HBZ-Tg) mice, in which HBZ is expressed under the control of the CD4 promoter/enhancer/silencer, so that only CD4^+^ T cells express HBZ [19, 32]. We also generated tax transgenic (tax-Tg) mice using the same promoter [33]. We isolated CD4^+^ T cells from HBZ-Tg and tax-Tg mice and evaluated their proliferation upon anti-CD3 stimulation. CD4^+^ T cells of HBZ-Tg mice proliferated much more than those of non-transgenic (non-Tg) mice, and the proliferation of CD4^+^ T cells was slightly enhanced in tax-Tg mice (Fig 1A and 1B). Co-culture of CD4^+^ T cells with dendritic cells (DC) further enhanced this proliferation (Fig 1A and 1B). However, the difference in proliferation between cells from HBZ-Tg and non-Tg mice was not observed in the presence of anti-CD28 antibody (0.3 μg/mL) (Fig 1C), indicating that CD4^+^ T cells of HBZ-Tg mice are hypersensitive to signaling via the TCR/CD3 complex.

To investigate whether the proliferation of CD4^+^ T cells of HBZ-Tg mice is increased *in vivo*, we induced experimental allergic encephalomyelitis (EAE) in HBZ-Tg and non-Tg mice by immunization with myelin oligodendrocyte glycoprotein (MOG)/complete Freund's adjuvant. Although disease severity was not different between HBZ-Tg mice and non-Tg mice (S1 Fig), the number of CD4^+^ T cells was increased only in the immunized HBZ-Tg mice compared with non-immunized HBZ-Tg and non-Tg mice (Fig 1D), suggesting that HBZ-expressing T cells have higher susceptibility to immune stimulation *in vivo*. HBZ-Tg did not show higher susceptibility to EAE regardless of impaired Treg function by HBZ [32]. It is speculated that partial inhibition of Treg functions by HBZ is not enough to increase incidence of EAE.

### Expression of co-stimulatory and co-inhibitory receptors on HBZ-expressing T cells and ATL cells

**Fig 2:**
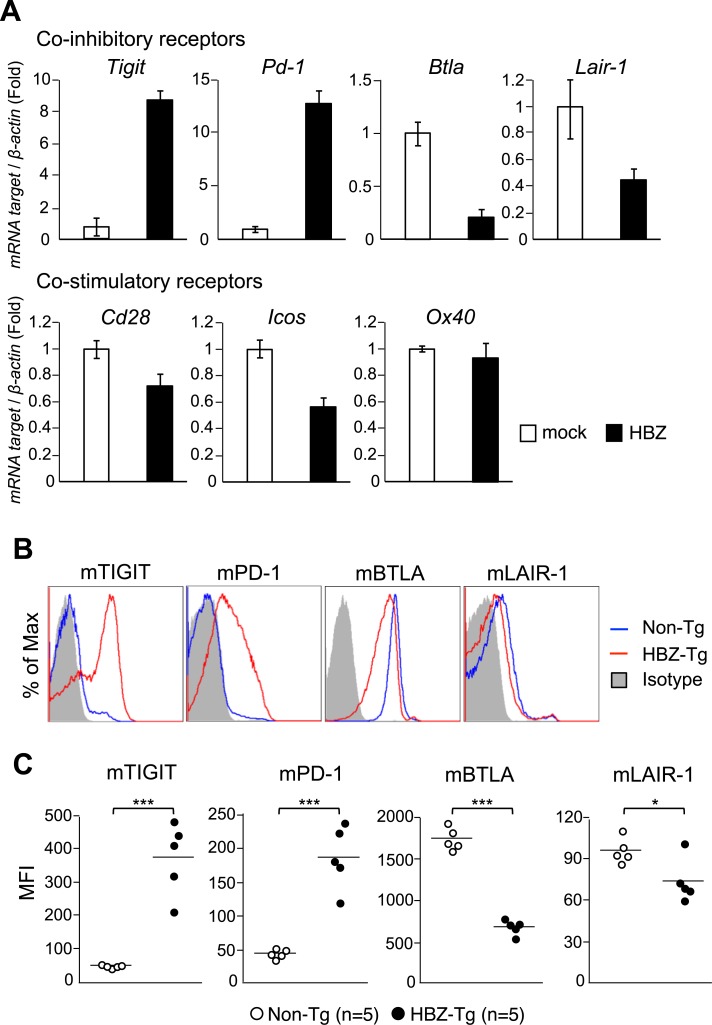
Expression of co-inhibitory receptors. (A) Transcripts of co-inhibitory and co-stimulatory receptor genes were quantified in HBZ transduced mouse T cells by real-time RT-PCR. (B) Splenocytes of non-Tg or HBZ-Tg mice (11 weeks old) were stained with anti-CD4, TIGIT, PD-1, BTLA and LAIR-1 antibodies. The expression of co-inhibitory receptors in CD4^+^ T cells was analyzed by flow cytometry. (C) The mean fluorescence intensity (MFI) of TIGIT, PD-1, BTLA and LAIR-1 in CD4^+^ T cells of non-Tg (n = 5) and HBZ-Tg (n = 5) mice is shown.

It has been reported that HTLV-1 infected cells and ATL cells express both co-stimulatory (OX40) and co-inhibitory receptors (PD-1 and TIGIT) on their surfaces [23, 25, 34, 35]. These findings suggest that HTLV-1 influences expression of co-inhibitory and co-stimulatory receptors. Therefore, we analyzed their expressions in HBZ-expressing T cells by real-time RT-PCR. As shown in Fig 2A, the expression of the co-inhibitory receptors *TIGIT* and *PD-1* was enhanced, whereas transcription of other co-inhibitory receptors, *BTLA* and *Lair-1*, was suppressed in HBZ transduced T cells. HBZ suppressed somewhat the transcription of the co-stimulatory receptors *CD28* and *ICOS* but did not influence that of *OX40*. In accordance with this finding, flow cytometric analyses showed that in CD4^+^ T cells from HBZ-Tg mice, cell-surface PD-1 and TIGIT were enhanced, while BTLA and LAIR-1 were decreased (Fig 2B and 2C). HBZ changed the cell-surface expression of co-stimulatory receptors only slightly or not at all (S2 Fig). On the other hand, expression of co-inhibitory receptors did not differ on CD4^+^ T cells between tax-Tg and non-Tg mice (S3 Fig). Expression of *HBZ* and *tax* in these transgenic mice was confirmed by RT-PCR (S4 Fig).

**Fig 3:**
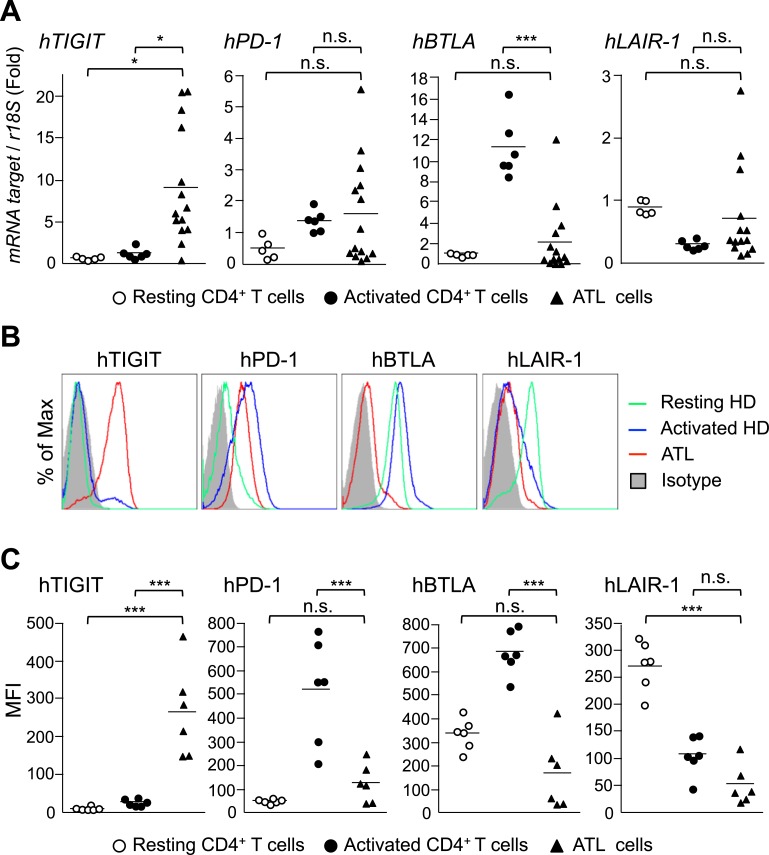
Expression of TIGIT, PD-1, BTLA and Lair-1 in ATL cases. (A) Relative mRNA expression levels of co-inhibitory receptors in resting healthy donor CD4^+^ T cells (n = 5), PHA-activated CD4^+^ T cells (n = 5) and ATL cells (n = 14) were evaluated by real-time RT-PCR. (B) Cells from the same groups were stained with anti-CD4, TIGIT, PD-1, BTLA and LAIR-1 antibodies. Expression of co-inhibitory receptors on CD4^+^ T cells was analyzed by flow cytometry. (C) The MFI of TIGIT, PD-1, BTLA and LAIR-1 on the cells are shown.

To study whether similar changes in levels of these co-stimulatory and co-inhibitory receptors are observed in ATL cells, we analyzed transcription and cell surface expression of these co-receptors. As shown in Fig 3A and 3C, *TIGIT* transcription and expression were significantly increased in ATL cases. *PD-1* expression was upregulated in some ATL cases as reported previously [24]. *BTLA* transcription and cell-surface expression were not different in ATL cases compared with resting T cells, but suppressed compared with activated T cells (Fig 3A and 3C). Cell-surface expression of LAIR-1 was also suppressed in ATL cells. Transcripts of the *HBZ* and *tax* genes were measured by real-time RT-PCR in these cases (S5 Fig).

It makes sense that HBZ might decrease the expression of BTLA and LAIR-1, thus impairing their suppressive function and enhancing the proliferation of infected cells. However, enhanced expression of PD-1 and TIGIT would augment the suppressive function of these co-inhibitory receptors, leading to decreased proliferation of cells. This idea is not consistent with the observation that HBZ enhances proliferation of expressing T cells. Therefore, we speculated that even though HBZ increases the *expression* of TIGIT and PD-1, it may inhibit their suppressive *function*. On the other hand, expression of co-stimulatory receptors, ICOS and OX40 was decreased in ATL cases compared with control activated CD4^+^ T cells (S6 Fig).

### HBZ confers resistance to the suppressive effect of co-inhibitory receptors

**Fig 4:**
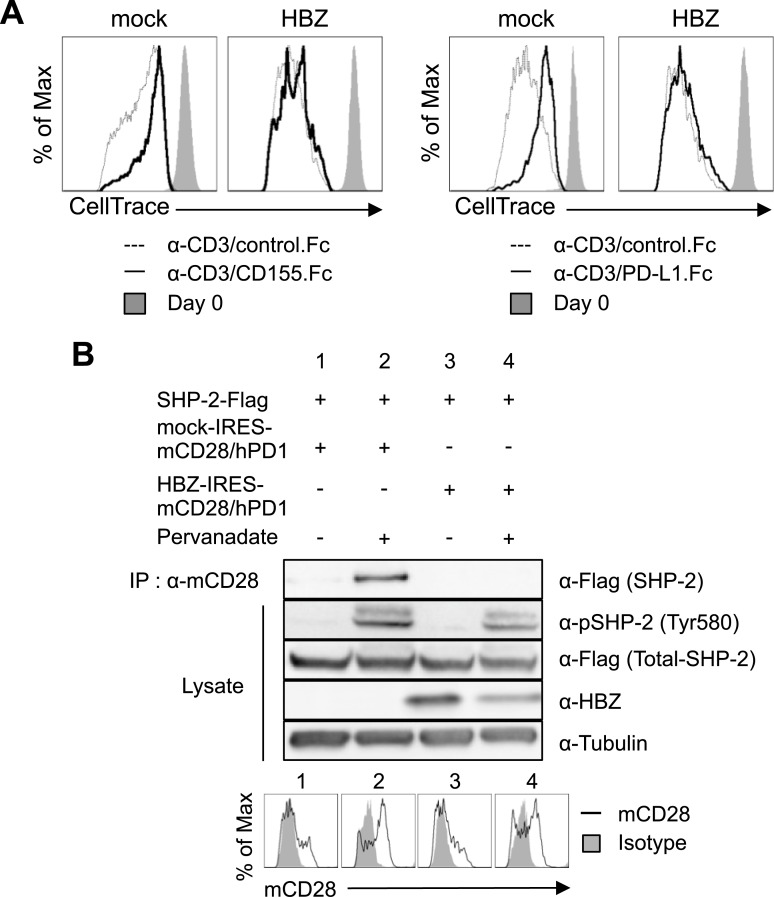
HBZ inhibits the suppressive effects of PD-1 and TIGIT. (A) HBZ-transduced murine primary CD4^+^ T cells were labeled with 5 μM CellTrace Violet and stimulated with anti-CD3/CD155.Fc-coated beads, anti-CD3/PD-L1.Fc-coated beads, or anti-CD3/control.Fc-coated beads at a bead-to-cell ratio of 1:1 for three days. CellTrace Violet dilution was analyzed by flow cytometry. (B) SHP-2 and PD-1 interaction in the presence or absence of HBZ was analyzed by immunoprecipitation. Vectors expressing SHP-2-Flag and HBZ (or mock)-IRES-mCD28/hPD1 were transfected into 293FT cells. After 48 hours, transfected cells were stimulated with pervanadate for 5 min and cell lysates were immunoprecipitated with anti-mCD28 antibody.

The co-inhibitory receptors PD-1 and TIGIT possess ITIM or ITSM domains, and inhibit cell proliferation [21]. As described above, we hypothesized that HBZ may interfere with the T-cell inhibitory function induced by PD-1/PD-L1 and/or TIGIT/CD155 interaction. To study this possibility, we next analyzed the suppressive activity of TIGIT/CD155 interaction in the presence or absence of HBZ. CD4^+^ T cells were transduced with retroviruses expressing HBZ and stimulated with anti-CD3/CD155.Fc-coated beads or anti-CD3/control.Fc-coated beads. We then measured proliferation of the cells. As shown in Fig 4A, interaction with anti-CD3/CD155.Fc-coated beads suppressed the proliferation of CD4^+^ T cells transduced with empty vector, but not those transduced with *HBZ*, indicating that HBZ impairs TIGIT/CD155 mediated growth inhibition. Likewise, HBZ interfered with the suppressive effect of PD-1/PD-L1 interaction (Fig 4A). These data suggest that HBZ targets a common molecule(s) that is involved in mediating suppressive signals from both PD-1 and TIGIT. Furthermore, suppressive signal through BTLA was also inhibited by the presence of HBZ (S7 Fig). Thus, HBZ not only suppresses BTLA expression but also functionally inhibits suppressive signaling from BTLA.

Inhibitory signals through the ITIM and ITSM motifs of PD-1 and TIGIT are mediated by SHP-1 and SHP-2 [21, 36, 37], negative regulators of TCR signaling that dephosphorylate ZAP-70 and CD3ζ and suppress T-cell activation [38, 39]. We analyzed whether HBZ influences the interaction between the intracytoplasmic region of PD-1 and SHP-2. To study the interaction of SHP-2 with the cytoplasmic region of PD-1, we generated a chimeric molecule in which the intracytoplasmic region of human PD-1 was fused to the transmembrane and extracytoplasmic regions of mouse CD28 (mCD28/hPD1) [40]. Pervanadate induces tyrosine phosphorylation of intracellular proteins including PD-1, thus recruiting SHP-2 to the ITSM motif [40]. After treatment with pervanadate, SHP-2 was recruited to the chimeric molecule (Fig 4B, lane 2), while HBZ inhibited this interaction (lane 4). Thus, HBZ hinders recruitment of SHP-2 to the ITSM motif of PD-1.

**Fig 5:**
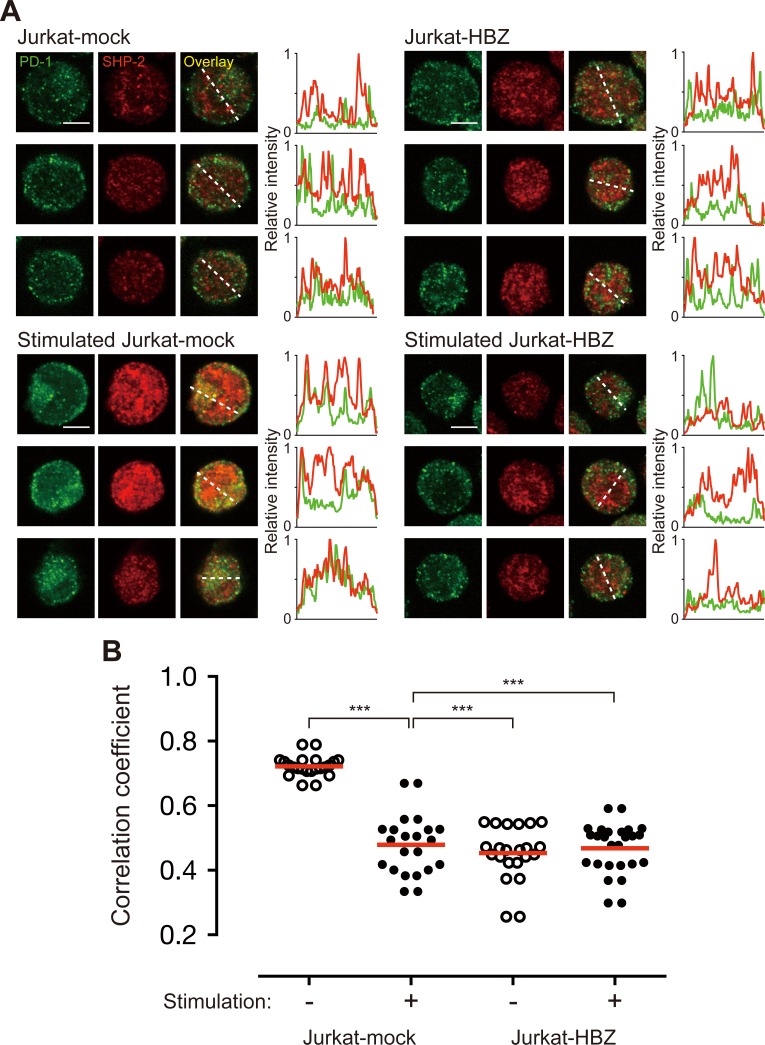
HBZ inhibits co-localization of PD-1 and SHP-2. (A) Staining of unstimulated or pervanadate-stimulated Jurkat-mock and Jurkat-HBZ cells was performed using antibodies against PD-1 (green) and SHP-2 (red). All scale bars are 5 μm. Three representative images derived from each sample are shown. Relative fluorescence intensities of PD-1 (green line) and SHP-2 (red line) were obtained on the white dotted line. (B) Co-localization of PD-1 and SHP-2 was judged by Pearson’s correlation coefficient between green and red channels. Each circle represents an individual cell. Statistical analysis was performed using one-way ANOVA with Tukey’s post hoc test.

Binding of PD-L1 induces a transient PD-1-TCR co-localization within microclusters–a co-localization that transiently associates with SHP-2 [41]. After stimulation by pervanadate, PD-1 formed TCR microclusters in Jurkat cells as reported previously (S8 Fig). Next, we analyzed the co-localization of PD-1 and SHP-2 after treatment with pervanadate to observe whether HBZ inhibits the interaction between PD-1 and SHP-2. As shown in Fig 5A, SHP-2 co-localized with PD-1 in Jurkat cells after pervanadate stimulation (stimulated Jurkat-mock). However, co-localization of these molecules was suppressed by the presence of HBZ (stimulated Jurkat-HBZ).

To quantitatively analyze the co-localization of PD-1 and SHP-2, we visualized PD-1 and SHP-2 using confocal microscopy. Captured raw images were analyzed using ImageJ software with the JACoP plug-in, and Pearson’s correlation coefficient [an index for the relationship of green (PD-1) and red (SHP-2) pixels] was calculated (Fig 5B). Co-localization of PD-1 and SHP-2 in Jurkat mock-transfected cells was minimal without pervanadate stimulation (the mean correlation coefficient value was 0.45), but these two molecules were highly co-localized upon pervanadate stimulation (correlation coefficient of 0.72). This value decreased dramatically, to 0.48, in the presence of HBZ (Jurkat-HBZ), indicating that HBZ strongly interferes with the co-localization of PD-1 and SHP-2. These data show that HBZ inhibits recruitment of SHP-2 to the cytoplasmic region of PD-1.

### HBZ suppresses phosphorylation of SHP-2 and dephosphorylation of ZAP-70

**Fig 6:**
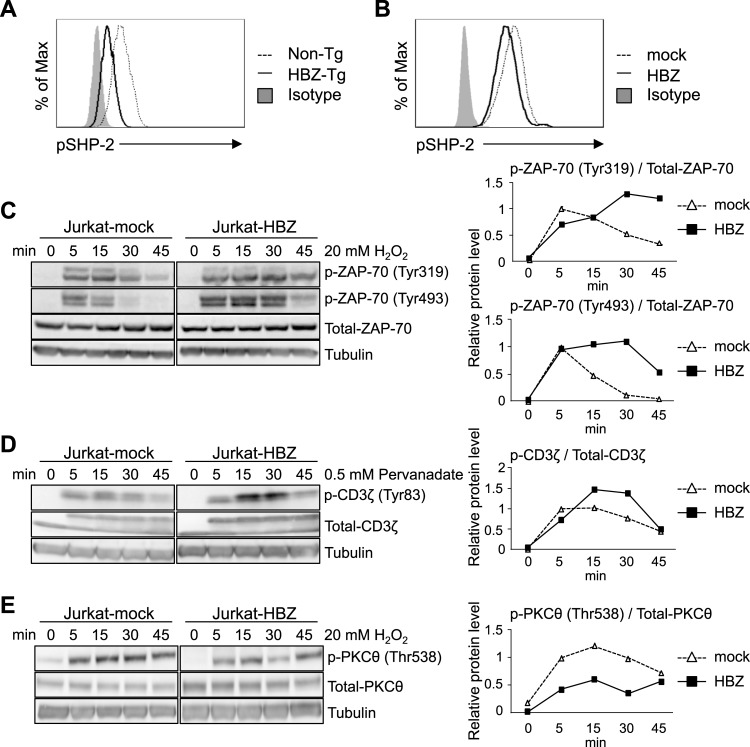
Effect of HBZ on phosphorylation of SHP-2, ZAP-70, CD3ζ and PKCθ. (A) Phosphorylation levels of SHP-2 (Tyr580) in CD4^+^ T cells of non-Tg and HBZ-Tg mice were analyzed by flow cytometry. Splenocytes of non-Tg or HBZ-Tg mice (8 weeks old) were stained with anti-CD4 and pSHP-2 (Tyr580) antibodies. (B) The phosphorylation level of SHP-2 (Tyr580) of HBZ-transduced murine primary CD4^+^ T cells was analyzed by flow cytometry. After HBZ transduction, cells were stimulated with anti-CD3/PD-L1.Fc-coated beads at bead-to-cell ratio of 1:1 for 6 hours. (C-E) Dephosphorylation of ZAP-70, CD3ζ and PKCθ was analyzed by immunoblotting (left). Jurkat-mock and Jurkat-HBZ cells were stimulated with 20 mM H_2_O_2_ or 0.5 mM pervanadate for 0, 5, 15, 30 or 45 min. Phosphorylation levels were also analyzed by densitometry (right). Relative protein levels were calculated as the ratio of phosphorylated protein to total protein.

As shown above, HBZ inhibits the interaction between SHP-2 and PD-1. Indeed, phosphorylation of SHP-2 (Tyr580) was decreased in CD4^+^ T cells of HBZ-Tg mice (Fig 6A) and in HBZ-transduced murine primary T cells (Fig 6B). SHP-2 functions to dephosphorylate ZAP-70 and CD3ζ. After induction of phosphorylation by H_2_O_2_ [42], tyrosine phosphorylation of ZAP-70 lasted for a longer time in the presence of HBZ (Fig 6C). Similarly, tyrosine phosphorylation of CD3ζ persisted longer after it was induced by pervanadate (Fig 6D). These data indicate that HBZ interferes with the function of SHP-2, leading to suppressed dephosphorylation of ZAP-70 and CD3ζ.

PD-1 suppresses T-cell proliferation not only by interacting with SHP-1 and SHP-2, but also by interacting with PKCθ. Threonine phosphorylation (T538) of PKCθ is associated with its activation and IL-2 production by T cells. Signaling via PD-1 inhibits PKCθ T538 phosphorylation [43]. As shown in Fig 6E, HBZ did not enhance phosphorylation of PKCθ T538, in contrast to ZAP-70 and CD3ζ. These results suggest that HBZ mediated activation of TCR signaling is mainly through inhibition of the tyrosine phosphatase, SHP-2.

### HBZ interacts with THEMIS

**Fig 7:**
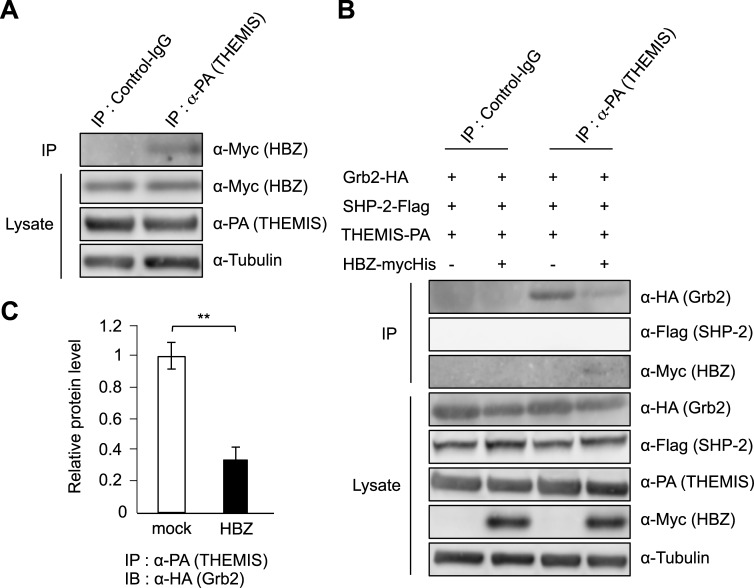
Interaction of HBZ with SHP associated factors. (A) Interaction between HBZ and THEMIS was analyzed by immunoprecipitation. (B) Interaction between THEMIS and, Grb2 or SHP-2, or HBZ was analyzed by immunoprecipitation. (A, B) Vectors expressing THEMIS, Grb2, SHP-2 and HBZ were transfected into 293FT cells (3.5×10^6^ cells, 10 cm dish). After 48 hours, transfected cells were stimulated with H_2_O_2_ for 5 min and cell lysates were immunoprecipitated with anti-PA antibody or normal rat IgG as a control. (C) THEMIS and Grb2 interaction in the presence or absence of HBZ was also analyzed with a densitometer. Relative protein levels were calculated as the ratio of immunoprecipitated protein to total protein.

This study shows that HBZ inhibits the recruitment of SHP-2 to the cytoplasmic region of PD-1. However, it remains unknown how HBZ interacts with the complex containing SHP-2. Recently, THEMIS has been reported to interact with Grb2 and SHP-1 or 2, and inhibit T-cell activation [44]. We analyzed the interaction of HBZ with these host factors and found that HBZ binds to THEMIS, but not to Grb2 and SHP-2 (Fig 7A and S9 Fig), suggesting that THEMIS is a target of HBZ. Next, we analyzed whether HBZ affects the interaction between THEMIS and Grb2. We confirmed that THEMIS interacts with Grb2, and found that this interaction is hindered by the presence of HBZ (Fig 7B and 7C). These data demonstrate that HBZ interacts with THEMIS and partially impairs the association of THEMIS with Grb2. This interaction of HBZ with the complex containing SHP may hinder recruitment of SHP to the ITSM and ITIM motifs of co-inhibitory receptors such as PD-1.

**Fig 8:**
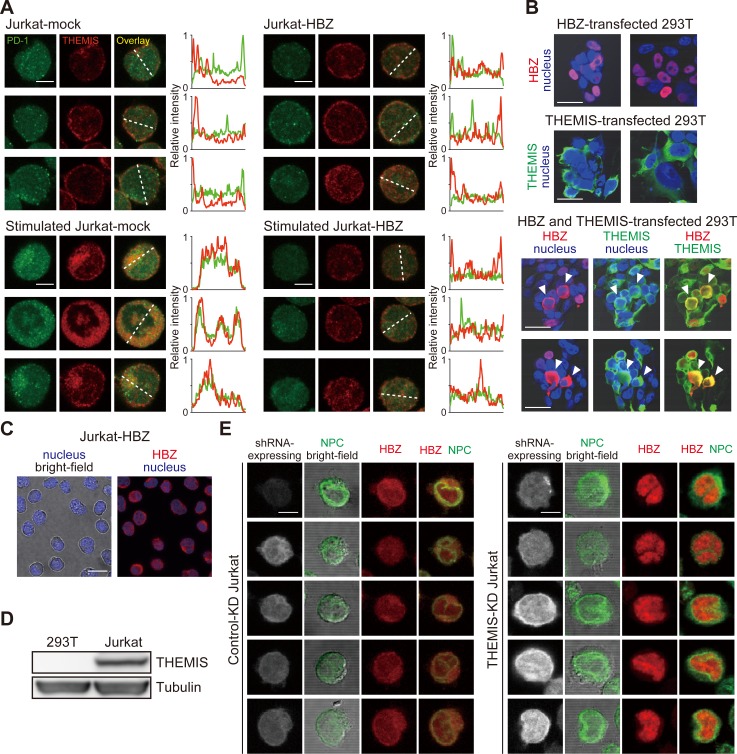
Subcellular localization of HBZ and THEMIS. (A) Staining of unstimulated or pervanadate-stimulated Jurkat-mock and Jurkat-HBZ cells was performed using antibodies against PD-1 (green) and THEMIS (red). All scale bars are 5 μm. Three representative images derived from each sample are shown. Relative fluorescence intensities of PD-1 (green line) and THEMIS (red line) were obtained over white dotted line. (B) Localizations of HBZ (red) and THEMIS (green) were analyzed in HBZ and/or THEMIS-transfected 293T cells. Nucleus was stained with DAPI (blue). Co-localization of HBZ and THEMIS was found in co-transfected cells (indicated by arrowheads). Two representative images derived from each sample are shown. All scale bars are 30 μm. (C) Localization of HBZ in Jurkat-HBZ cells was detected using anti-HBZ antibody. Nucleus was stained with DAPI (blue). Scale bar is 10 μm. (D) Expression of THEMIS was detected by immunoblotting in 293T cells and Jurkat cells. (E) Effect of THEMIS on the localization of HBZ was analyzed in myc-tagged HBZ-introduced THEMIS-KD and luciferase-KD (served as a control) Jurkat cells. shRNA-expressing cells express GFP as a marker (shown in gray). Cells were stained with antibodies against nuclear pore complex (NPC; green) and myc-tag (HBZ; red). Five representative images derived from each sample are shown. All scale bars are 5 μm.

Next, we analyzed whether HBZ interferes co-localization of PD-1 and THEMIS in the T cells. Stimulation by pervanadate induced co-localization of PD-1 and THEMIS, which was inhibited by HBZ (Fig 8A). Thus, HBZ interacts with THEMIS, which perturbs the complex containing SHP and impairs suppressive signal from PD-1 or TIGIT. To check whether suppressed THEMIS enhances T-cell proliferation through disrupted negative signal, we inhibited THEMIS expression by shRNA and found that suppressed THEMIS expression decreased T-cell proliferation (S10 Fig). It has been reported that proliferation of T cells from THEMIS knockout mice was suppressed [45], suggesting that THEMIS is also critical for T-cell proliferation in addition to suppressive signaling from co-inhibitory receptors.

THEMIS interacts with ITIM or ITSM domain of PD-1 and TIGIT in the cytoplasm whereas it has been reported that HBZ is primarily localized in the nucleus [46]. Therefore, localization of HBZ was analyzed in the presence of THEMIS. As reported previously, THEMIS existed in the cytoplasm (50 of 50 cells: 100%) whereas HBZ was mainly localized in the nucleus of 293T cells (67 of 74 cells: 90.5%)(Fig 8B). When both proteins were expressed, HBZ was co-localized with THEMIS in the cytoplasm (28 of 79 cells: 35.4%)(Fig 8B). Thus, THEMIS shifted localization of HBZ from nucleus to cytoplasm in 293T cells. When we analyzed localization of HBZ in T cells, HBZ was detected in both the nucleus and the cytoplasm (Fig 8C). Then, we analyzed THEMIS expression in 293T and Jurkat cells, and found that only Jurkat cells expressed THEMIS (Fig 8D), suggesting that THEMIS is responsible for cytoplasmic localization of HBZ.

**Fig 9:**
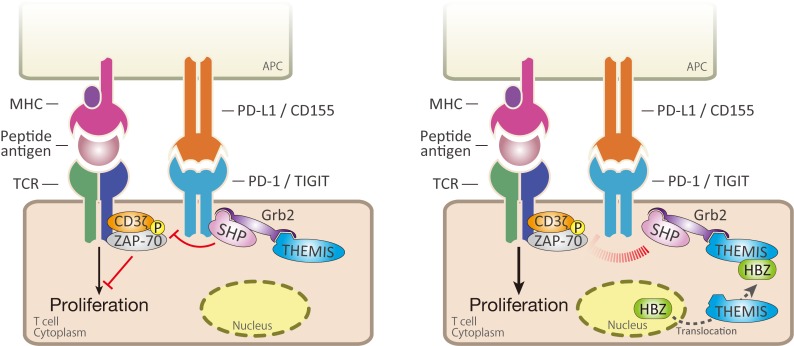
Schema of HBZ mediated inhibition of inhibitory signaling from co-inhibitory receptors. After T-cell activation through TCR, PD-1 forms microcluster with TCR complex. Complex containing SHP-2, Grb2 and THEMIS recruits to ITSM or ITIM motif of co-inhibitory receptors, PD-1 and TIGIT. SHP-1 dephosphorylates critical molecules for T-cell activation including ZAP-70 and CD3ζ. HBZ interacts with THEMIS, which changes subcellular localization of HBZ. HBZ interferes complex formation of THEMIS, Grb2 and SHP-2, which results in inhibition of suppressive signal from co-inhibitory receptors, and enhanced activation of T cells.

To clarify the localization of HBZ in T cells in detail, we detected HBZ using antibody to the nuclear pore complex, and confirmed that HBZ was present in both nucleus and cytoplasm (Fig 8E). To study whether cytoplasmic localization of HBZ is attributed to THEMIS, we suppressed THEMIS expression using shRNA, and found that HBZ was present largely in the nucleus, suggesting that endogenous THEMIS contributes to changed localization of HBZ from the nucleus to the cytoplasm (Fig 9).

## Discussion

Co-stimulatory and co-inhibitory receptors control T-cell function and determine T-cell fate after a T cell is stimulated by TCR signaling [21]. In this study, we showed that HBZ enhances the susceptibility of expressing T cells to TCR-mediated signaling by perturbing signaling from co-inhibitory receptors. This study is the first to demonstrate that HBZ targets various co-inhibitory receptors by different mechanisms, and enhances proliferation. Expression of BTLA and LAIR-1 is decreased by HBZ, while HBZ impairs the suppressive function of PD-1 and TIGIT through inhibited recruitment of the SHP-2 containing complex to the cytoplasmic domain of PD-1. In contrast to BTLA and LAIR-1, expression of PD-1 and TIGIT are in fact upregulated by HBZ. Why does HBZ enhance expression of TIGIT and PD-1 among co-inhibitory receptors? Increased TIGIT expression competes with CD226, a co-stimulatory receptor, for binding with CD155, resulting in inhibition of T-cell activation [47]. In addition, HBZ suppressed CD226 expression [25]. Furthermore, our previous study indicated that TIGIT expressed on T cells is implicated in immune suppression through enhanced production of IL-10 from T cells and DC by reverse signaling [25]. Since reverse signal from PD-L1 and L2 on DC is also associated with suppressive phenotype of DC and moderate increase in IL-10 expression [48], PD-1 on T cells is also implicated in immune suppression. DC expresses the TIGIT ligand, CD155, and PD-1 ligands, PD-L1 and PD-L2, on the surface. DC-T-cell interaction plays a key role in immune responses to viral infections [49]. We have reported that increased expression of TIGIT on HBZ expressing T cells induces IL-10 production [25]. Furthermore, IL-12p40 production of DC cells was severely impaired in HBZ-Tg mice, which is likely caused by TIGIT on T cells [25, 47]. Thus HBZ suppresses host immune responses through enhanced PD-1 and TIGIT expression while simultaneously impairing SHP-2 mediated inhibitory signaling from these co-inhibitory receptors. In other words, HBZ modifies the functions of the co-inhibitory receptors PD-1 and TIGIT to allow the virus to evade the host immune system.

SHP-1 and 2 form complexes with Grb2 and THEMIS in T cells, and inhibit TCR mediated signaling [44]. Knockdown of THEMIS increased TCR-induced CD3ζ phosphorylation, a phenomenon that resembles the changes caused by HBZ. In this study, we show that HBZ interacts with THEMIS and weakens the interaction between THEMIS and Grb2. These data suggest that HBZ binding to THEMIS hinders recruitment of this complex to the ITSM motif of PD-1 and thus impedes suppressive signals. THEMIS is expressed only in the T-cell lineage [44, 50]. Therefore, it is thought that HBZ may not inhibit the co-inhibitory signal in non-T cells. Since the receptor for HTLV-1 is glucose transporter 1, HTLV-1 infects a variety of cells *in vivo* [51]. However, only infected T cells proliferate *in vivo*. Our observation that HBZ binds to THEMIS and impairs the growth-suppressive signal might account for this T-cell specificity of HTLV-1.

TCF-1 and LEF-1, transcription factors of classical Wnt signaling pathway, are critical for T-cell development in the thymus [52], and their expressions are suppressed in peripheral memory T cells. We have reported that TCF-1 and LEF-1 inhibit function of Tax, which may critically influence the peripheral T-cell tropism of this virus [53]. It remains unknown how this virus specifically promotes proliferation of infected mature T cells. This study reveals that interaction between HBZ and THEMIS impedes suppressive signal by interfered recruitment of SHP-2 to ITSM motif in T cells. This is thought to be a mechanism of T-cell specificity in HTLV-1 induced proliferation. Thus, HBZ and Tax determine specificity of HTLV-1 infected cells.

Analysis of the transcriptomes of 57 ATL cases using RNA-seq identified fusion gene products that contained five CD28-related in-frame fusions (CTLA4-CD28 or ICOS-CD28) that likely induce continuous or prolonged CD28 co-stimulatory signaling [54]. In addition, amplification of CD28 was frequently detected in ATL cases. Thus the CD28 co-stimulatory molecule is a frequent target of somatic changes in ATL cells. As shown in this study, HBZ perturbs co-inhibitory signaling, which enhances proliferation of not only ATL cells but also other HTLV-1 infected cells. Thus, in ATL cells, TCR signaling is a target of both somatic mutation and HBZ. We assume that HBZ perturbs TCR signaling and promotes proliferation beginning in individuals at the carrier state, and then somatic mutations potentiate and exacerbate the proliferative responses.

Recently it has been reported that structural variations in the 3’ untranslated region of PD-L1 enhanced PD-L1 expression in various cancer cells [55]. In particular, these structural variations were frequently observed in ATL (27% of 49 ATL cases). Overexpression of PD-L1 on ATL cells inhibits immune attack from CD8^+^ T cells through interaction with PD-1. Since ATL cells also express PD-1, PD-L1 might suppress the proliferation of ATL cells. However, since HBZ interrupts the suppressive signal from PD-1 through inhibited recruitment of the SHP containing complex to the ITSM motif, ATL cells avoid growth suppression while maintaining the immune suppressive effects of PD-1/PD-L1 interaction on CD8^+^ T cells.

HBZ is primarily localized in the nucleus [46], and interacts with transcription factors, which include p65, Smad3, and c-Jun, and other host factors such as p300 in the nucleus [56–59]. However, it has been reported that HBZ interacts with GADD34 in the cytoplasm, which activates the mammalian target of rapamycin (mTOR) signaling [60]. This finding suggests that HBZ also functions in the cytoplasm by interacting with host factors. This study showed that interaction of HBZ with THEMIS changes its localization and HBZ functions in the cytoplasm, and HBZ interferes suppressive function of co-inhibitory receptors. Thus, HBZ exerts the functions in both nucleus and cytoplasm.

In this study, we demonstrate that HBZ inhibits suppressive signaling from co-inhibitory receptors by decreased transcription or by inhibition of recruitment of SHP-2. Furthermore, HBZ enhances the expression of TIGIT and PD-1, which are associated with immune suppression. Thus, HBZ enables expressing T cells to survive and proliferate *in vivo* by utilizing and modifying the functions of co-inhibitory receptors.

## Materials and Methods

### Mice

C57BL/6J mice were purchased from CLEA Japan. Transgenic mice expressing *HBZ* (HBZ-Tg mice) under the control of the murine CD4-specific promoter/enhancer/silencer have been described previously [19]. All HBZ-Tg mice were heterozygotes for the transgene. Transgenic mice expressing tax (tax-Tg mice) under the control of the same promoter were generated as reported [33].

### Cells

Jurkat cell line was provided by Dr. S. Sakaguchi (Osaka University, Japan). Jurkat cell lines stably expressing the spliced form of HBZ (Jurkat-HBZ) and control (Jurkat-mock) cells were cultured in RPMI 1640 medium supplemented with 10% fetal bovine serum (FBS) and 1 mg/mL G418 (Nacalai Tesque, Kyoto, Japan) [61]. The 293T cell line was purchased from ATCC (Manassas, VA, USA) and cultured in Dulbecco’s modified Eagle medium (DMEM) supplemented with 10% FBS. The 293FT cell line was purchased from Life Technologies and cultured in DMEM supplemented with 10% FBS and 0.5 mg/mL G418. The packaging cell line, Plat-E, was provided by Dr. T. Kitamura (Institute of Medical Science, The University of Tokyo, Japan) and cultured in DMEM containing 10% FBS, 10 μg/mL blasticidin and 1 μg/mL puromycin. These cell lines were grown at 37°C under a 5% CO_2_ atmosphere.

### Clinical samples

Peripheral blood mononuclear cells (PBMCs) of ATL patients and healthy donors were collected by Ficoll-Paque PLUS (GE Healthcare, Little Chalfont, UK). CD4^+^ T cells of healthy donors were isolated by Human CD4^+^ T Cell Enrichment Cocktail (STEMCELL Technologies, Vancouver, Canada) according to the manufacturer’s instructions. To obtain activated CD4^+^ T cells, CD4^+^ T cells were stimulated by 10 μg/mL phytohemagglutinin (PHA) (Sigma-Aldrich, St. Louis, MO, USA) for three days.

### Ethics statement

Animal experiments were performed in strict accordance with the Japanese animal welfare bodies (Law No. 105 dated 19 October 1973, modified on 2 June 2006), and the Regulation on Animal Experimentation at Kyoto University. The protocol was approved by the Institutional Animal Research Committee of Kyoto University (permit numbers D13-02, D14-02, D15-02, and A10-3). Experiments using clinical samples were conducted according to the principles expressed in the Declaration of Helsinki, and approved by the Institutional Review Board of Kyoto University (permit number G310). ATL patients and healthy blood donors provided written informed consent for the collection of samples and subsequent analysis.

### Plasmids

pMX-IG, pMX-HBZ-IG and pMX-BTLA-IG were used for retrovirus production. The coding sequence of BTLA was amplified from cDNA of a wild type C57BL/6J mouse, and subcloned into pMX-IG. The HBZ expression vector, pcDNA3.1 HBZ-mycHis, was described previously [58]. The SHP-2 expression vector, pSP65SRa-SHP2-Flag, was kindly given by Dr. M. Hatakeyama (The University of Tokyo, Japan). The entire coding regions of Grb2 and THEMIS were amplified from cDNA prepared from resting human PBMCs or Jurkat cells. These PCR fragments were subcloned into pCMV-HA (Clontech Laboratories, Palo Alto, CA, USA) or pCAGGS-PA. The resulting plasmids were designated pCMV-HA-Grb2 and pCAGGS-PA-THEMIS, and they express HA (YPYDVPDYA)-tagged Grb2 and PA (GVAMPGAEDDVV)-tagged THEMIS, respectively. For generation of the chimeric mCD28/human PD-1 (hPD-1) expression vector, the mCD28 extracellular and transmembrane domains (bases 9–617 of NM_007642) were amplified from cDNA prepared from stimulated murine T cells, and the hPD-1 intracellular domain (bases 645–935 of NM_005018) was amplified from cDNA prepared from PHA-stimulated human PBMCs. These fragments were ligated and the resultant fragment was substituted for the GFP cording region of pMX-HBZ-IRES-GFP.

### Preparation of antibody-coupled beads

Anti-CD3 antibody (145-2X11, R&D systems, Minneapolis, MN, USA) together with recombinant mouse CD155.Fc or the control.Fc (Sino Biological, Beijing, China) or PD-L1. Fc or the control.Fc (R&D systems) or HVEM.Fc (R&D systems) were covalently attached to Dynabeads M450 Tosylactivated (Invitrogen, Thermo Fisher Scientific, Waltham, MA, USA). Anti-CD3 antibody together with control.Fc was used for the control. For each 10^7^ beads, 1 μg of anti-CD3 antibody (20% of total protein) and 4 μg of CD155.Fc, PD-L1.Fc, HVEM.Fc or control.Fc (80%) were used.

### Generation of THEMIS knock-down Jurkat cells

The *PiggyBac*-based shRNA expression vector, pB-CMV-GreenPuro-H1 (System Biosciences, Palo Alto, CA, USA), containing shRNA against THEMIS or luciferase (control), was introduced into Jurkat cells together with *PiggyBac* transposase expression vector by using Neon Transfection System (Invitrogen). Target sequences of each shRNA are shown in S1 Table.

### Cell proliferation assay

To measure the proliferation of CD4^+^ T cells of HBZ-Tg mice, we isolated murine splenic CD4^+^ T cells using the CD4 T Lymphocyte Enrichment Set (BD Biosciences, San Jose, CA, USA). Murine splenic dendritic cells were isolated from collagenase-digested low-density cells using the Dendritic Cell Enrichment Set (BD Biosciences). Purified CD4^+^ T cells were labeled with 5-(and-6)-carboxyfluorescein diacetate succinimidyl ester (CFSE, Molecular Probes, Thermo Fisher Scientific, Waltham, MA, USA). Labeled CD4^+^ T cells of HBZ-Tg, non-Tg and tax-Tg mice (2×10^5^ cells/well) were cultured with or without dendritic cells (1×10^4^ cells/well) from non-Tg mice for three days with soluble anti-CD3 antibody (30 ng/mL) stimulation in round-bottomed 96-well plates. CFSE dilution was analyzed by flow cytometry. For TIGIT/CD155 and PD-1/PD-L1 proliferation assays, HBZ or empty vector transduced cells (see below) were labeled with CellTrace Violet (Invitrogen) and stimulated with anti-CD3/CD155.Fc or anti-CD3/PD-L1.Fc or anti-CD3/control.Fc-coated beads at a bead-to-cell ratio of 1:1 for three days. Dye dilution was analyzed by flow cytometry.

### Retroviral transduction

Transfection of the packaging cell line, Plat-E, was performed as reported [62]. Murine CD4^+^ T cells were activated by immobilized anti-CD3 (1 μg/mL) and soluble anti-CD28 (0.1 μg/mL) in 12-well plates. After 24 hours, activated T cells were transduced with virus supernatant and 4 μg/mL polybrene, and centrifuged at 3,000 rpm for 60 min. Cells were subsequently cultured for 48 hours.

### Real-time RT-PCR

Total RNA was isolated using Trizol Reagent (Invitrogen) and treated with DNase I to remove the genomic DNA. cDNAs were synthesized from 1 μg of total RNA using random primer and SuperScript III or IV reverse transcriptase according to the manufacturer’s instructions (Invitrogen). mRNA expression was analyzed by real-time PCR using FastStart Universal SYBR Green Master (Roche Diagnostics, Basel, Switzerland) and the StepOnePlus Real-Time PCR System (Applied Biosystems, Thermo Fisher Scientific, Waltham, MA, USA) according to the manufacturer’s instructions. Primers used in this study are shown in S1 Table.

### Flow cytometric analysis

The following antibodies were used for flow cytometric analyses. Anti-mCD4 (GK1.5), mTIGIT (IG9), mCD28 (E18), mICOS (C398.4A), mOX40 (OX-86), hCD4 (RPA-T4), hPD-1 (29F.1A12), hBTLA (MIH26), hLAIR-1 (NKTA255), hCD28 (CD28.2), hICOS (C398.4A) and hOX40 (Ber-ACT35) antibodies were all purchased from BioLegend (San Diego, CA, USA). Anti-mBTLA (6F7), mLAIR-1 (113) and hTIGIT (MBSA43) antibodies were purchased from eBioscience (San Diego, CA, USA). Anti-mPD-1 (J43) was from BD Pharmingen (BD Biosciences). Anti-phospho-SHP-2 (Tyr580) antibody was from Cell Signaling Technology (Danvers, MA, USA). Anti-mouse IgG1, mouse IgG2b and rat IgG1 (BioLegend), Armenian hamster IgG (eBiosciences) and mouse IgG2a (BD Pharmingen) were purchased for isotype controls. For detection of SHP-2 phosphorylation, cells were permeabilized using BD Phosflow perm buffer II (BD Biosciences) according to the manufacturer’s instructions. Flow cytometric analysis was carried out using a FACSVerse with FACSuite software (BD Biosciences) and FlowJo (TreeStar, Ashland, OR, USA).

### Experimental autoimmune encephalomyelitis (EAE)

For the induction of EAE, five to seven-week old HBZ-Tg or control non-transgenic (non-Tg) C57BL/6J mice were immunized with an emulsion containing MOG peptide 35–55 (MEVGWYRSPFSRVVHLYRNGK). The emulsion was prepared by sonication, mixing 1 mL of MOG solution (1 mg/mL in PBS) with 1 mL of complete Freund’s adjuvant (Difco Laboratories, Detroit, MI, USA) containing desiccated *Mycobacterium butyricum*. The emulsion was injected subcutaneously in the area near the axillary lymph nodes and on both sides at the base of the tale of each mouse (50 μL/site, a total of 4 sites/mouse). On days 0 and 2 post-immunization, mice were injected intraperitoneally with 50 μL of a Pertussis toxin (Kaketsuken, Kumamoto, Japan) solution (4 μg/mL). Thereafter, mice were monitored daily for clinical signs of encephalomyelitis. A clinical score was assigned according to the following criteria: 0, no symptoms; 1, mild limp tail; 1.5, limp tail; 2, unilateral hind limb weakness or abnormal gait; 2.5, unilateral hind limb paralysis or bilateral hind limb weakness; 3, paraplegia; 3.5, unilateral fore limb weakness, with paraplegia; 4, unilateral fore limb paralysis or bilateral fore limb weakness; 4.5, bilateral fore limb paralysis; 5, moribund or dead.

### Immunoprecipitation

For the immunoprecipitation studies of SHP-2 and PD-1, 293FT cells were transfected with the indicated expression vectors using Lipofectamine LTX (Invitrogen) according to manufacturer’s instructions. After 48 hours, cells were stimulated with 0.1 mM pervanadate solution for 5 min. The cell lysates were immunoprecipitated for 60 min at 4°C with 5 μg of anti-mCD28 (37.51), and immune complexes were incubated with Protein G-Sepharose (GE Healthcare) for 60 min at 4°C. For the immunoprecipitation studies of THEMIS, Grb2, SHP-2 and HBZ, 293FT cells were transfected as described above. After 48 hours, cells were stimulated with H_2_O_2_ for 5 min. The cell lysates were immunoprecipitated with 20 μg of anti-PA (NZ-1), anti-HA (HA-7) or anti-Flag (M2) antibodies, and immune complexes were incubated as described above. Normal mouse and rat IgG (Santa Cruz Biotechnology, Dallas, TX, USA) were used as controls.

### Immunoblotting

The following antibodies were used for immunoblotting: anti-phospho-SHP-2 (Tyr580), phospho-ZAP-70 (Tyr319 and Tyr493), ZAP-70 (99F2), phospho-PKCθ (Thr538) and PKCθ (E1I7Y) antibodies were purchased from Cell Signaling Technology. Anti-phosphor-CD3ζ (Tyr83), CD3ζ and anti-THEMIS were purchased from Abcam (Cambridge, UK). Anti-PA (NZ-1) was purchased from Wako, Osaka, Japan. Anti-Flag-HRP (M2), HA-HRP (HA7), Myc (9E10), and tubulin (DM1A) antibodies were purchased from Sigma-Aldrich. Anti-mouse IgG-HRP, rabbit IgG-HRP and rat IgG-HRP antibodies were purchased from GE Healthcare. Mouse anti-HBZ monoclonal antibody (clone 1A10) was generated by immunizing C57BL/6 with using keyhole limpet hemocyanin (KLH)-conjugated HBZ peptide 97–133 (CKQIAEYLKRKEEEKARRRRRAEKKAADVARRKQEEQE).

### Immunofluorescence analysis

To detect co-localizations of PD-1 with SHP-2, THEMIS or TCR, Jurkat-mock or Jurkat-HBZ cells were stimulated with 0.2 mM pervanadate solution for 2 min at 37°C. To evaluate the effect of THEMIS on the localization of HBZ, THEMIS-knocked down (KD) and control (luciferase KD) Jurkat cells were transfected with pcDNA 3.1 HBZ-mycHis or empty vector. The cells were washed with PBS and placed on MAS-coated glass slides (Matsunami Glass, Osaka, Japan). To detect HBZ, 293T cells cultured on type I collagen (Cellmatrix, Nitta Gelatin, Osaka, Japan)-coated coverslips, were transfected with pcDNA 3.1 HBZ-mycHis and/or pCAGGS-PA-THEMIS. The cells were fixed with 4% paraformaldehyde for 15 min, permeabilized with 0.2% Triton X-100 for 15 min, and blocked by incubation in 5% donkey serum (Jackson ImmunoResearch, West Grove, PA, USA) for 60 min. For immunostaining, the cells were incubated with anti-SHP-2 (sc-280), anti-PD-1 (sc-10297), anti-TCR β (sc-5277) (all Santa Cruz Biotechnology), anti-THEMIS (ab126771), anti-Nuclear Pore Complex Proteins (ab24609) (all Abcam), anti-myc (9E10, Sigma-Aldrich or Abcam) or anti-PA (NZ-1) antibodies for 60 min, followed by incubation with Alexa Fluor 488-conjugated donkey anti-goat IgG, Alexa Fluor 488-conjugated donkey anti-rat IgG, Alexa Fluor 594-conjugated donkey anti-mouse IgG, Alexa Fluor 594-conjugated donkey anti-rat IgG, Alexa Fluor 647-conjugated donkey anti-mouse IgG or Alexa Fluor 647-conjugated donkey anti-rabbit IgG antibodies (all Invitrogen), or DyLight 405-conjugated donkey anti-mouse IgG (Jackson ImmunoResearch) for 30 min. The stained cells were mounted with ProLong Gold Antifade Reagent or ProLong Gold Antifade Reagent with DAPI (all Molecular Probes, Thermo Fisher Scientific), imaged using an FV1000 confocal microscope (Olympus, Tokyo, Japan) or a Leica TCS SP8 (Leica Microsystems, Wetzlar, Germany), and analyzed with ImageJ.

### Statistical analyses

For Figs 1, 2, 7, S2 and S10, statistical significance was determined by the two-tailed unpaired Student’s t-test. For Figs 3, 5 and S6, statistical analysis was performed using the one-way ANOVA with Tukey’s post hoc test (GraphPad Prism, GraphPad Software, La Jolla, CA, USA). Asterisks indicate the statistical significance as follows: *P < 0.05; **P < 0.01; ***P < 0.001; n.s., not significant.

## Supporting Information

S1 Fig Severity of EAE did not differ between HBZ-Tg and non-Tg mice. EAE was induced in HBZ-Tg and control mice. These mice were monitored daily for symptoms, and clinical scores were determined. The graph shows the average values at the indicated time-points for the HBZ-Tg (n = 7) and the control non-Tg (n = 5) mice. The induction of EAE and determination of clinical scores are described in the Materials and Methods section.
(PPTX)

S2 Fig Expression of co-stimulatory receptors in CD4^+^ T cells of HBZ-Tg and non-Tg mice. (A) Splenocytes of non-Tg or HBZ-Tg mice (11 weeks old) were stained with anti-CD4, CD28, ICOS and OX40 antibodies. Expression of co-stimulatory receptors in CD4^+^ T cells was analyzed by flow cytometry. (B) MFI of CD28, ICOS and OX40 in CD4^+^ T cells of non-Tg (n = 5) and HBZ-Tg mice (n = 5).
(PPTX)

S3 Fig Expression of co-inhibitory receptors in CD4^+^ T cells of tax-Tg and non-Tg mice. Expression of co-inhibitory receptors (TIGIT, PD-1, BTLA and LAIR-1) was analyzed on CD4^+^ T cells from tax-Tg and non-Tg mice.
(PPTX)

S4 Fig Expression of *tax* or *HBZ* in CD4^+^ T cells of non-Tg, tax-Tg and HBZ-Tg mice. Transcripts of the *HBZ* and *tax* genes were detected by RT-PCR in CD4^+^ T cells from non-Tg, tax-Tg, and HBZ-Tg mice.
(PPTX)

S5 Fig Expression of *HBZ* and *tax* in ATL cells. Transcripts of the *HBZ* and *tax* genes were measured by real-time RT-PCR in ATL cases (n = 14) that were used in Fig 3.
(PPTX)

S6 Fig Expression of co-stimulatory receptors in CD4^+^ T cells of healthy donors and ATL patients. (A) Relative expression levels of co-stimulatory receptors on resting CD4^+^ T cells, activated CD4^+^ T cells and CD4^+^ T cells of ATL patients were evaluated by real-time RT-PCR. (B) Expression of the co-stimulatory receptors CD28, ICOS and OX40 on CD4^+^ T cells was analyzed by flow cytometry. (C) Expression of CD28, ICOS and OX40 in CD4^+^ T cells is shown.
(PPTX)

S7 Fig HBZ inhibits the suppressive effect of BTLA. BTLA-transduced murine primary CD4^+^ T cells of non-Tg or HBZ-Tg mice were labeled with 5 μM CellTrace Violet and stimulated with anti-CD3/HVEM.Fc-coated beads or anti-CD3/control.Fc-coated beads at a bead-to-cell ratio of 1:1 for three days. CellTrace Violet dilution was analyzed by flow cytometry.
(PPTX)

S8 Fig Co-localization of PD-1 and TCRβ after stimulation by pervanadate. Co-localization between PD-1 (green) and TCRβ (red) was analyzed in unstimulated and pervanadate-stimulated Jurkat-mock cells. All scale bars are 2 μm. Relative fluorescence intensities of PD-1 (green line) and TCRβ (red line) were obtained over white dotted line.
(PPTX)

S9 Fig HBZ does not interact with SHP-2 and Grb2. Interaction between HBZ with SHP-2 (A) or Grb2 (B) was analyzed by immunoprecipitation. Vectors expressing Grb2, SHP-2 and HBZ were transfected into 293FT cells (3.5×10^6^ cells, 10-cm dish). After 48 hours, transfected cells were stimulated with H_2_O_2_ for 5 min and cell lysates were immunoprecipitated with anti-Flag or anti-HA antibodies or normal rat IgG as a control.
(PPTX)

S10 Fig Effect of THEMIS knockdown in T cells. (A) THEMIS expression was measured in control Jurkat cells and THEMIS knockdown Jurkat cells by Western blot method. (B) The shRNA-expressing Jurkat cells were seeded into 96-well plates (1×10^4^ cells/well). Cell numbers of each shRNA-expressing Jurkat cells were counted in triplicate by Trypan blue dye exclusion method.
(PPTX)

S1 Table Oligonucleotide sequences. Primers and shRNA target sequences used in this study are shown.
(DOCX)
