# Correction: HTLV-1 bZIP Factor Enhances T-cell Proliferation by Impeding the Suppressive Signaling of Co-inhibitory Receptors

**DOI:** 10.1371/journal.ppat.1006228

**Published:** 2017-02-23

**Authors:** Haruka Kinosada, Jun-ichirou Yasunaga, Kazuya Shimura, Paola Miyazato, Chiho Onishi, Tomonori Iyoda, Kayo Inaba, Masao Matsuoka

Fig 5 is incorrect. Fig 5B was generated using a statistical software, GraphPad PRISM. The data points in Fig 5B were duplicated by human error, and in addition, the Jurkat-mock with stimulation (+) and without stimulation (-) data were switched. These errors do not influence the results and conclusion of this study. The authors have provided a corrected Fig 5 here.

**Fig 5:**
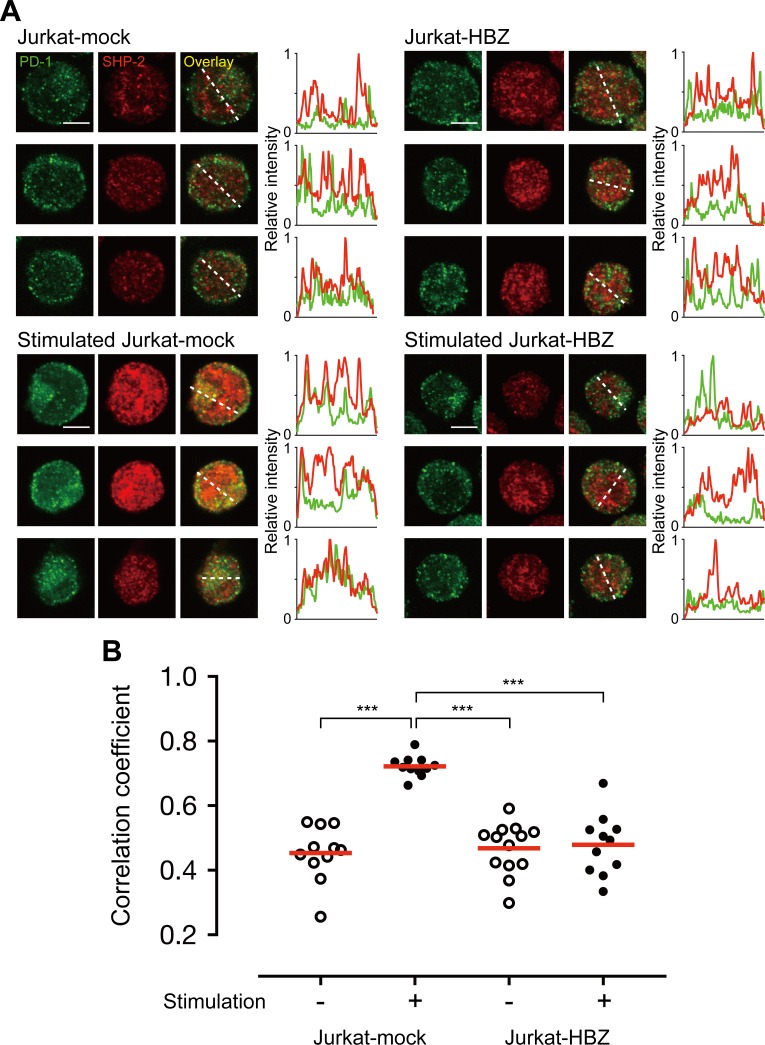
HBZ inhibits co-localization of PD-1 and SHP-2. **(**A) Staining of unstimulated or pervanadate-stimulated Jurkat-mock and Jurkat-HBZ cells was performed using antibodies against PD-1 (green) and SHP-2 (red). All scale bars are 5 μm. Three representative images derived from each sample are shown. Relative fluorescence intensities of PD-1 (green line) and SHP-2 (red line) were obtained on the white dotted line. (B) Co-localization of PD-1 and SHP-2 was judged by Pearson’s correlation coefficient between green and red channels. Each circle represents an individual cell. Statistical analysis was performed using one-way ANOVA with Tukey’s post hoc test.
