# Supplementary material for: HTLV-1 bZIP Factor Enhances T-Cell Proliferation by Impeding the Suppressive Signaling of Co-inhibitory Receptors
Source: PLoS Pathog. 2017 Jan 3;13(1):e1006120. doi: 10.1371/journal.ppat.1006120 (PMC5234849; doi:10.1371/journal.ppat.1006120)
Supplement: S1 Fig — EAE was induced in HBZ-Tg and control mice. These mice were monitored daily for symptoms, and clinical scores were determined. The graph shows the average values at the indicated time-points for the HBZ-Tg (n = 7) and the control non-Tg (n = 5) mice. The induction of EAE and determination of clinical scores are described in the Materials and Methods section. (PPTX) [file ppat.1006120.s001.pptx]

## Slide 1
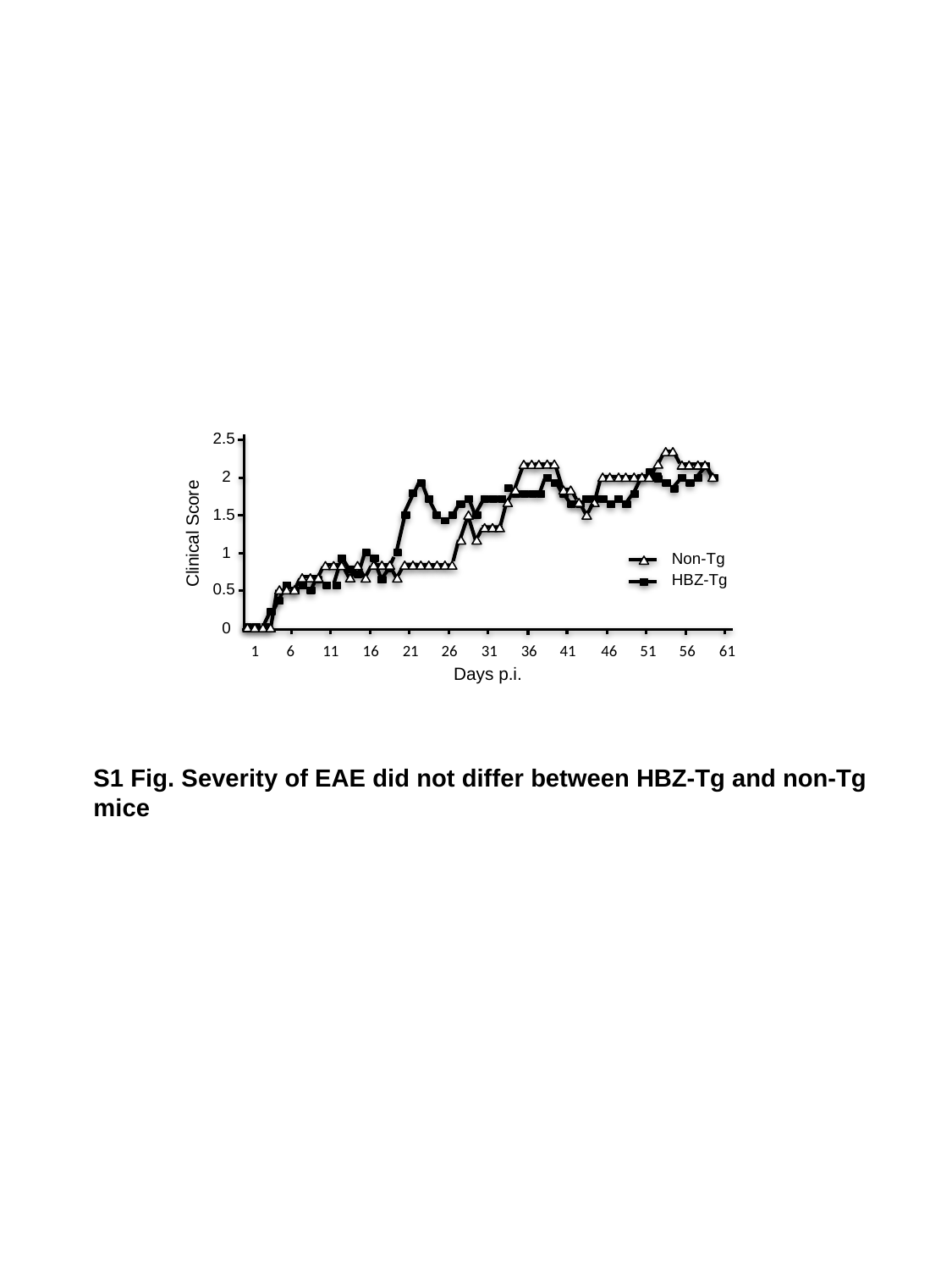

2.5
2
1.5
Clinical Score
1
Non-Tg
HBZ-Tg
0.5
0
1
6
11
16
21
26
31
36
41
46
51
56
61
Days p.i.
S1 Fig. Severity of EAE did not differ between HBZ-Tg and non-Tg mice
