# Supplementary material for: HTLV-1 bZIP Factor Enhances T-Cell Proliferation by Impeding the Suppressive Signaling of Co-inhibitory Receptors
Source: PLoS Pathog. 2017 Jan 3;13(1):e1006120. doi: 10.1371/journal.ppat.1006120 (PMC5234849; doi:10.1371/journal.ppat.1006120)
Supplement: S2 Fig — (A) Splenocytes of non-Tg or HBZ-Tg mice (11 weeks old) were stained with anti-CD4, CD28, ICOS and OX40 antibodies. Expression of co-stimulatory receptors in CD4+ T cells was analyzed by flow cytometry. (B) MFI of CD28, ICOS and OX40 in CD4+ T cells of non-Tg (n = 5) and HBZ-Tg mice (n = 5). (PPTX) [file ppat.1006120.s002.pptx]

## Slide 1
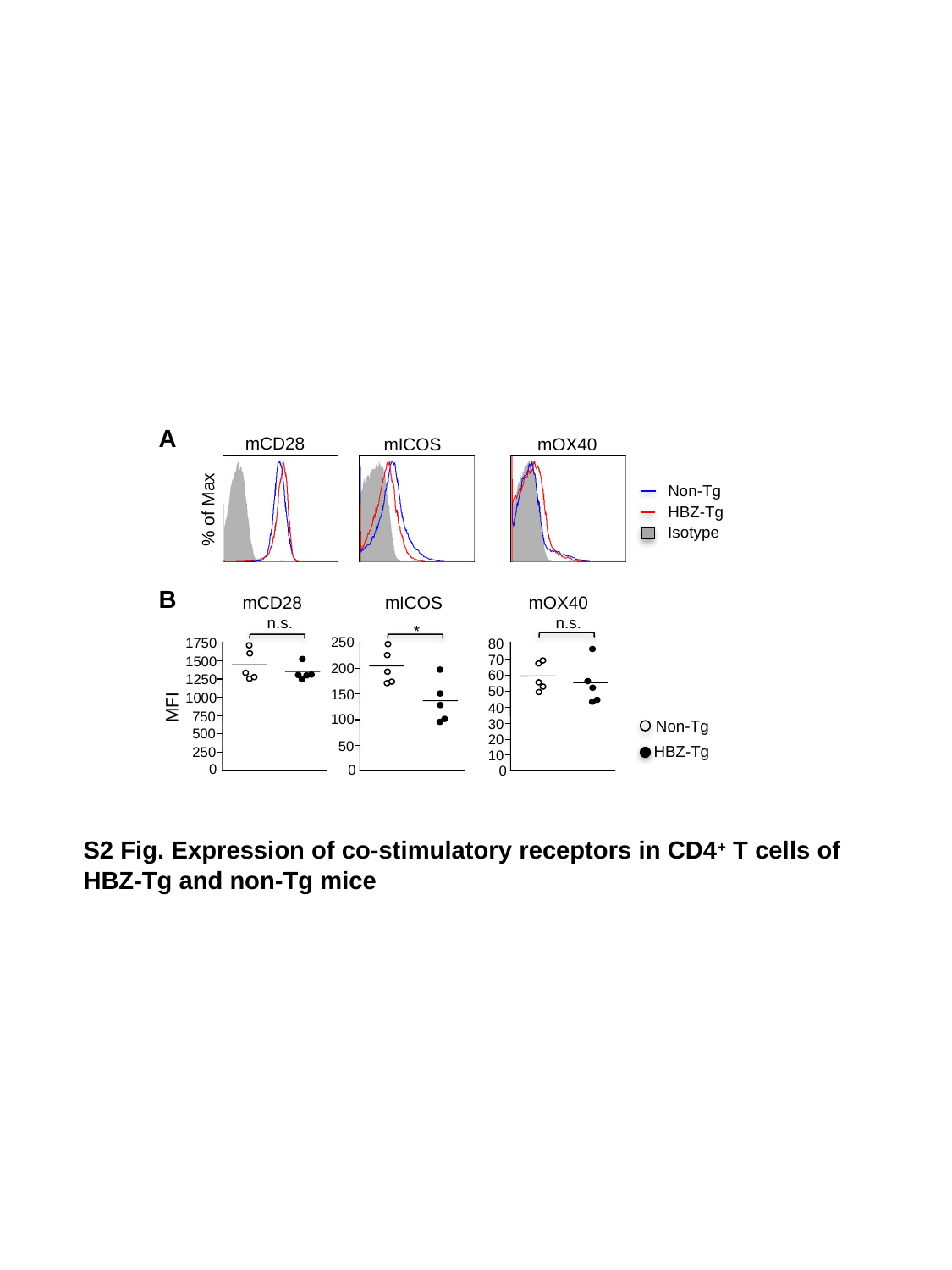

A
mCD28
mICOS
mOX40
Non-Tg
% of Max
HBZ-Tg
Isotype
B
mCD28
mICOS
mOX40
n.s.
n.s.
*
250
1750
80
70
1500
200
60
1250
50
150
1000
MFI
40
750
100
30
Non-Tg
500
20
50
HBZ-Tg
250
10
0
0
0
S2 Fig. Expression of co-stimulatory receptors in CD4+ T cells of HBZ-Tg and non-Tg mice
