# Supplementary material for: HTLV-1 bZIP Factor Enhances T-Cell Proliferation by Impeding the Suppressive Signaling of Co-inhibitory Receptors
Source: PLoS Pathog. 2017 Jan 3;13(1):e1006120. doi: 10.1371/journal.ppat.1006120 (PMC5234849; doi:10.1371/journal.ppat.1006120)
Supplement: S3 Fig — Expression of co-inhibitory receptors (TIGIT, PD-1, BTLA and LAIR-1) was analyzed on CD4+ T cells from tax-Tg and non-Tg mice. (PPTX) [file ppat.1006120.s003.pptx]

## Slide 1
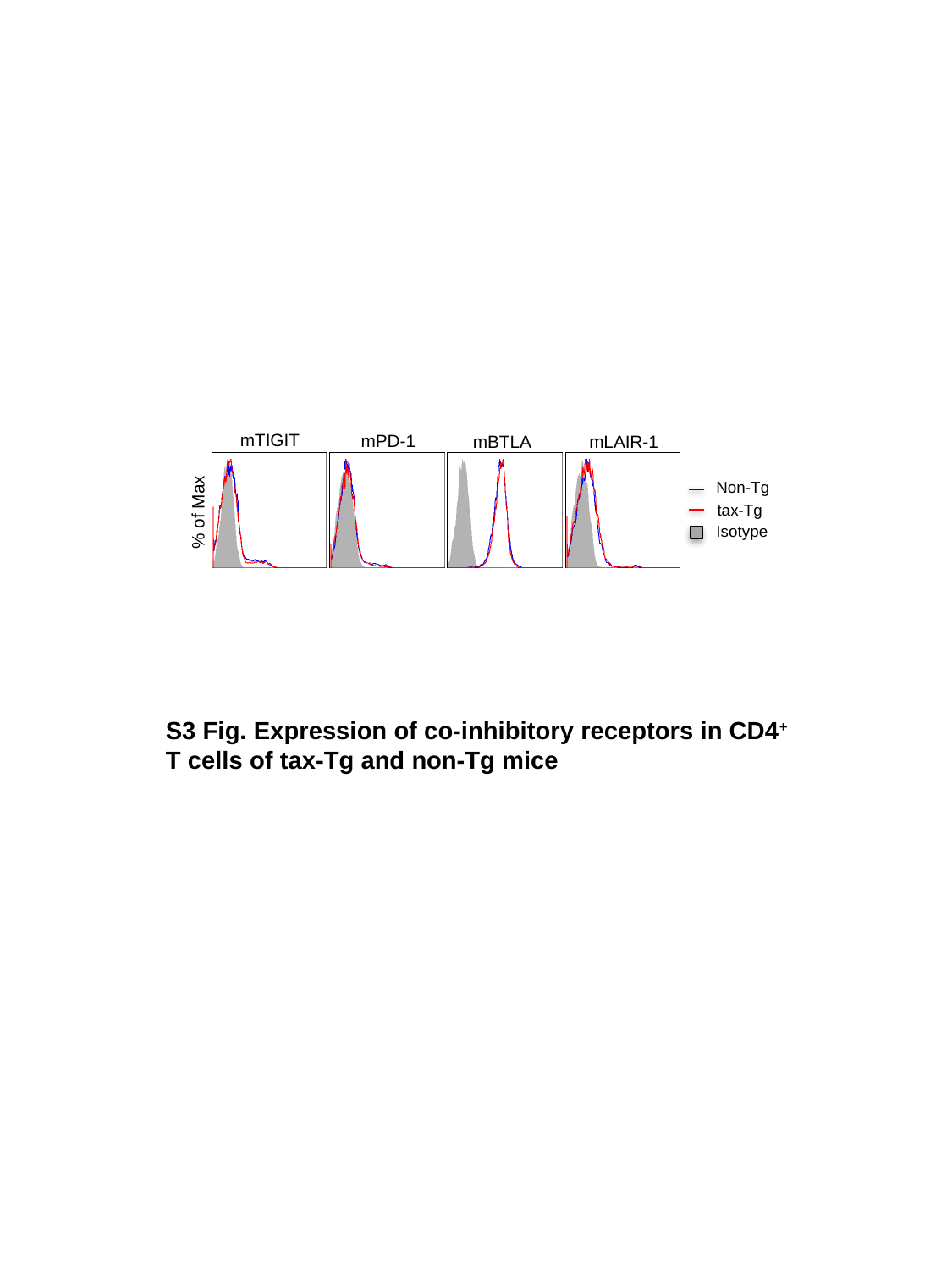

mTIGIT
mPD-1
mBTLA
mLAIR-1
Non-Tg
tax-Tg
Isotype
% of Max
S3 Fig. Expression of co-inhibitory receptors in CD4+ T cells of tax-Tg and non-Tg mice
