# Supplementary material for: HTLV-1 bZIP Factor Enhances T-Cell Proliferation by Impeding the Suppressive Signaling of Co-inhibitory Receptors
Source: PLoS Pathog. 2017 Jan 3;13(1):e1006120. doi: 10.1371/journal.ppat.1006120 (PMC5234849; doi:10.1371/journal.ppat.1006120)
Supplement: S4 Fig — Transcripts of the HBZ and tax genes were detected by RT-PCR in CD4+ T cells from non-Tg, tax-Tg, and HBZ-Tg mice. (PPTX) [file ppat.1006120.s004.pptx]

## Slide 1
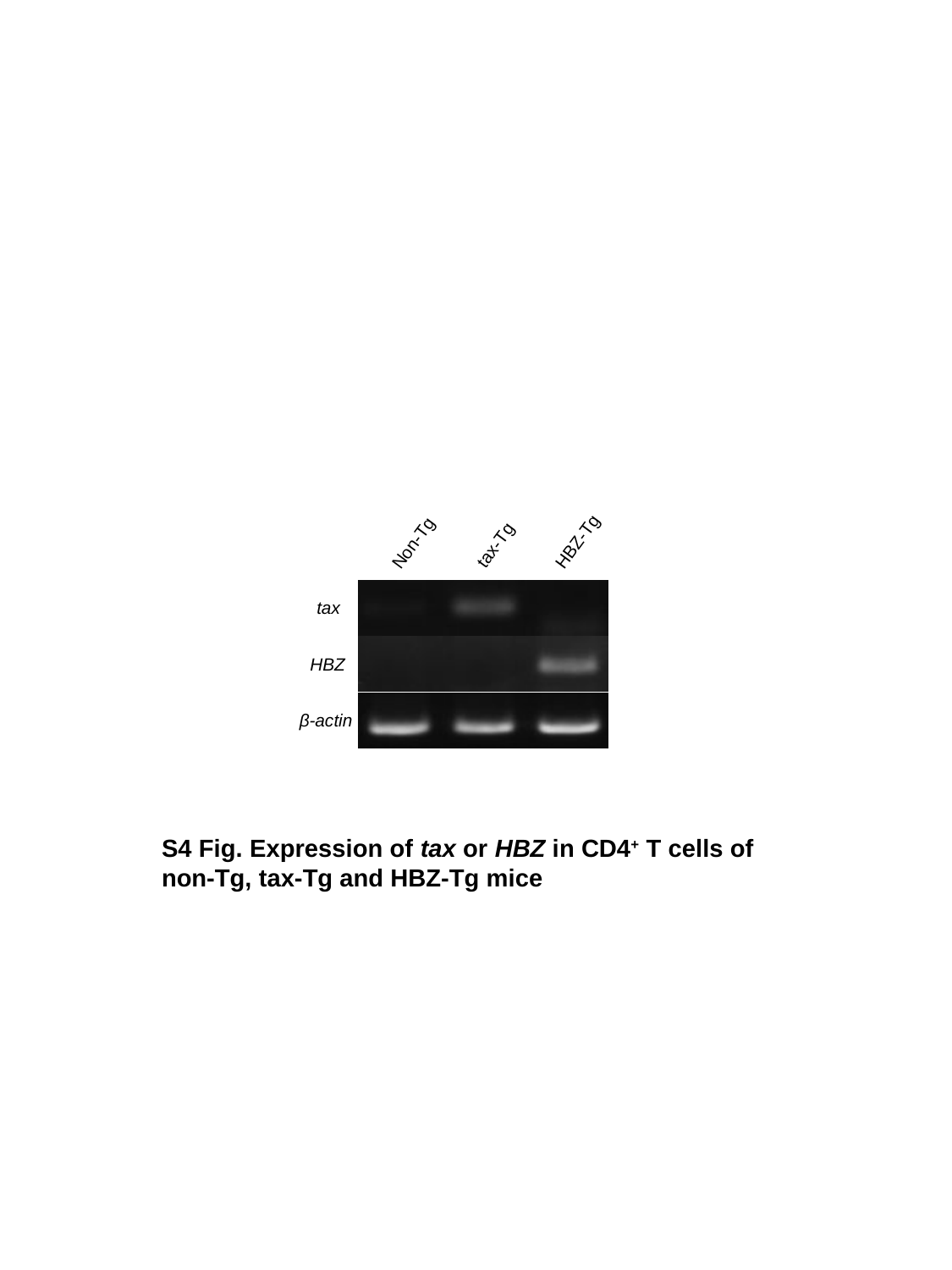

HBZ-Tg
Non-Tg
tax-Tg
tax
HBZ
β-actin
S4 Fig. Expression of tax or HBZ in CD4+ T cells of non-Tg, tax-Tg and HBZ-Tg mice
