# Supplementary figures and images for: HTLV-1 bZIP Factor Enhances T-Cell Proliferation by Impeding the Suppressive Signaling of Co-inhibitory Receptors
Source: PLoS Pathog. 2017 Jan 3;13(1):e1006120. doi: 10.1371/journal.ppat.1006120 (PMC5234849; doi:10.1371/journal.ppat.1006120)

## Slide 1
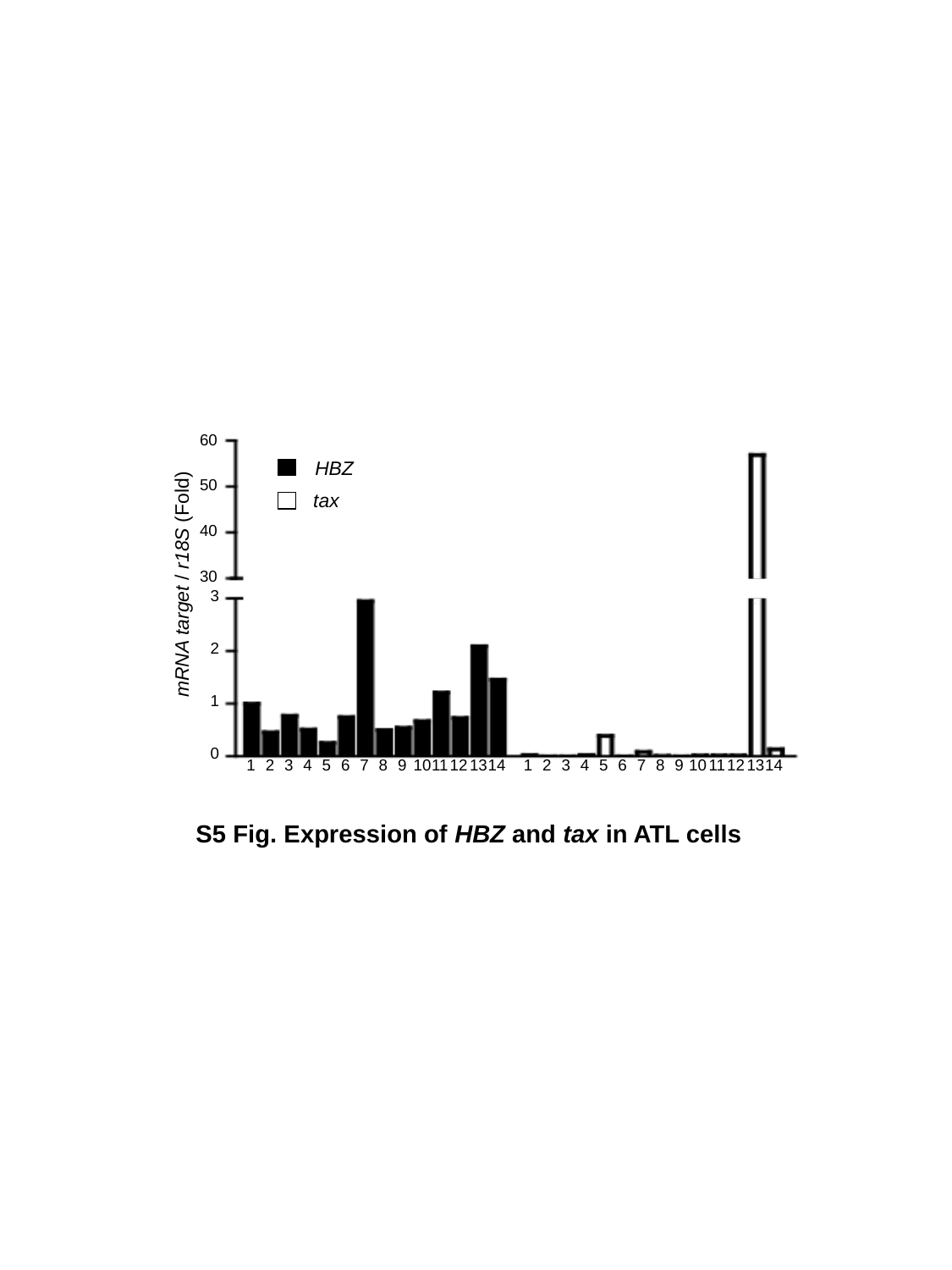

60
HBZ
tax
50
40
mRNA target / r18S (Fold)
30
3
2
1
0
1
2
3
4
5
6
7
8
9
11
12
13
14
1
2
3
4
5
6
7
8
9
10
11
12
13
14
10
S5 Fig. Expression of HBZ and tax in ATL cells

Supplement: S5 Fig — Transcripts of the HBZ and tax genes were measured by real-time RT-PCR in ATL cases (n = 14) that were used in Fig 3. (PPTX) [file ppat.1006120.s005.pptx]
