# Supplementary material for: HTLV-1 bZIP Factor Enhances T-Cell Proliferation by Impeding the Suppressive Signaling of Co-inhibitory Receptors
Source: PLoS Pathog. 2017 Jan 3;13(1):e1006120. doi: 10.1371/journal.ppat.1006120 (PMC5234849; doi:10.1371/journal.ppat.1006120)
Supplement: S6 Fig — (A) Relative expression levels of co-stimulatory receptors on resting CD4+ T cells, activated CD4+ T cells and CD4+ T cells of ATL patients were evaluated by real-time RT-PCR. (B) Expression of the co-stimulatory receptors CD28, ICOS and OX40 on CD4+ T cells was analyzed by flow cytometry. (C) Expression of CD28, ICOS and OX40 in CD4+ T cells is shown. (PPTX) [file ppat.1006120.s006.pptx]

## Slide 1
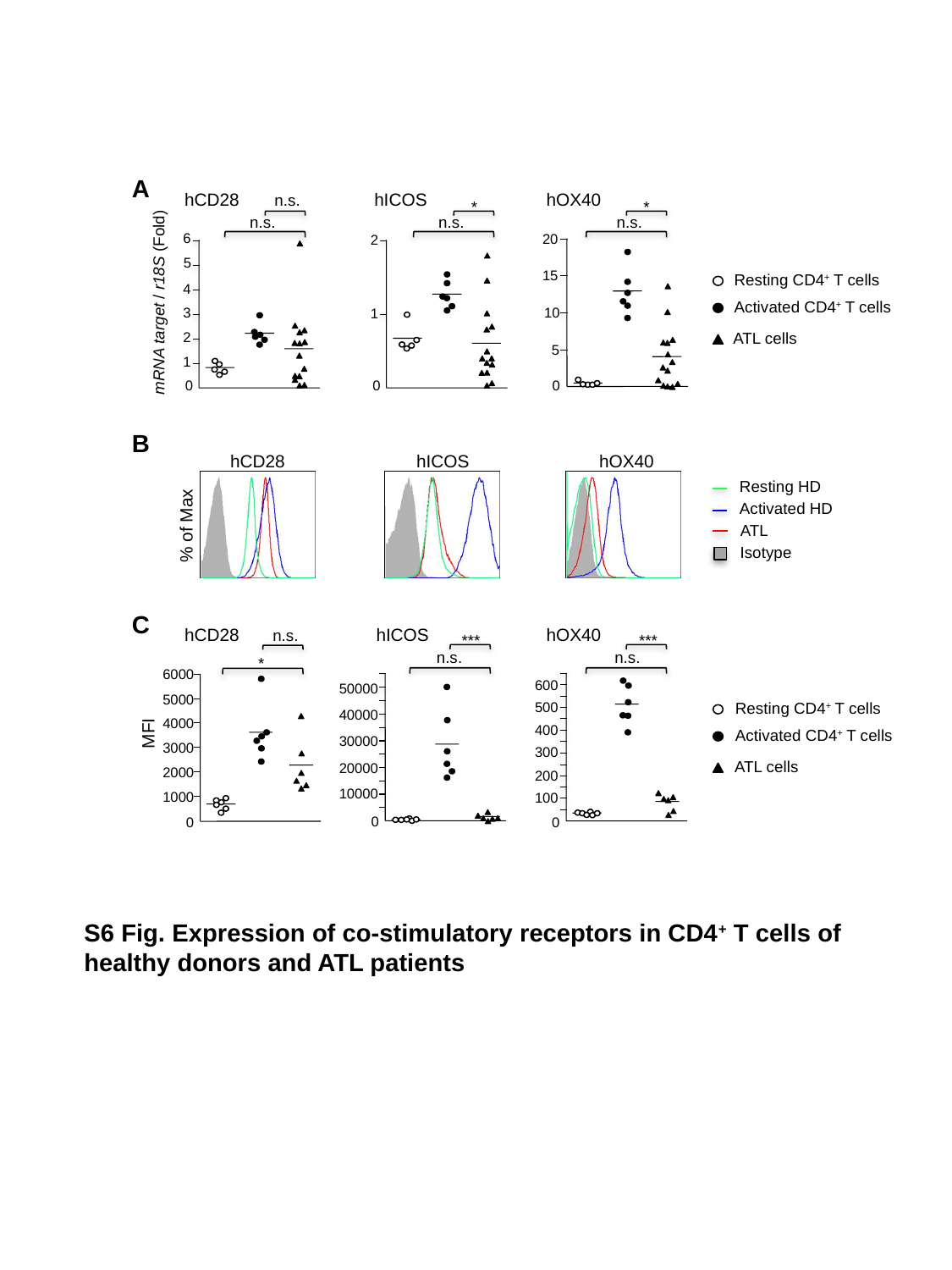

A
hCD28
hICOS
hOX40
n.s.
*
*
n.s.
n.s.
n.s.
6
20
2
5
15
Resting CD4+ T cells
Activated CD4+ T cells
ATL cells
4
mRNA target / r18S (Fold)
3
10
1
2
5
1
0
0
0
B
hCD28
hICOS
hOX40
Resting HD
Activated HD
% of Max
ATL
Isotype
C
hCD28
hICOS
hOX40
n.s.
***
***
n.s.
n.s.
*
6000
600
50000
5000
Resting CD4+ T cells
Activated CD4+ T cells
ATL cells
500
40000
4000
400
MFI
30000
3000
300
20000
2000
200
10000
1000
100
0
0
0
S6 Fig. Expression of co-stimulatory receptors in CD4+ T cells of healthy donors and ATL patients
