# Supplementary material for: HTLV-1 bZIP Factor Enhances T-Cell Proliferation by Impeding the Suppressive Signaling of Co-inhibitory Receptors
Source: PLoS Pathog. 2017 Jan 3;13(1):e1006120. doi: 10.1371/journal.ppat.1006120 (PMC5234849; doi:10.1371/journal.ppat.1006120)
Supplement: S7 Fig — BTLA-transduced murine primary CD4+ T cells of non-Tg or HBZ-Tg mice were labeled with 5 μM CellTrace Violet and stimulated with anti-CD3/HVEM.Fc-coated beads or anti-CD3/control.Fc-coated beads at a bead-to-cell ratio of 1:1 for three days. CellTrace Violet dilution was analyzed by flow cytometry. (PPTX) [file ppat.1006120.s007.pptx]

## Slide 1
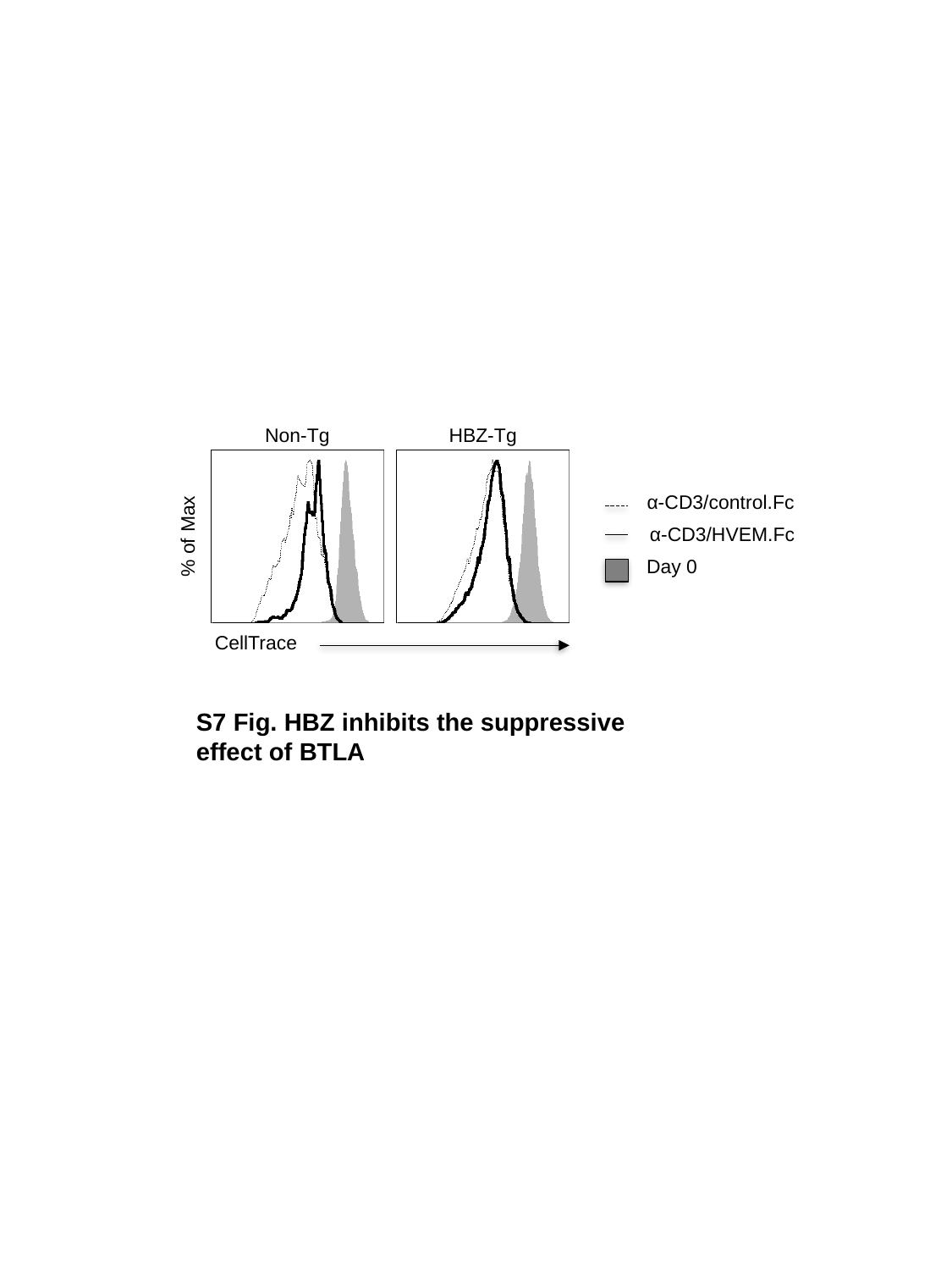

Non-Tg
HBZ-Tg
α-CD3/control.Fc
α-CD3/HVEM.Fc
Day 0
% of Max
CellTrace
S7 Fig. HBZ inhibits the suppressive effect of BTLA
