# Supplementary material for: HTLV-1 bZIP Factor Enhances T-Cell Proliferation by Impeding the Suppressive Signaling of Co-inhibitory Receptors
Source: PLoS Pathog. 2017 Jan 3;13(1):e1006120. doi: 10.1371/journal.ppat.1006120 (PMC5234849; doi:10.1371/journal.ppat.1006120)
Supplement: S8 Fig — Co-localization between PD-1 (green) and TCRβ (red) was analyzed in unstimulated and pervanadate-stimulated Jurkat-mock cells. All scale bars are 2 μm. Relative fluorescence intensities of PD-1 (green line) and TCRβ (red line) were obtained over white dotted line. (PPTX) [file ppat.1006120.s008.pptx]

## Slide 1
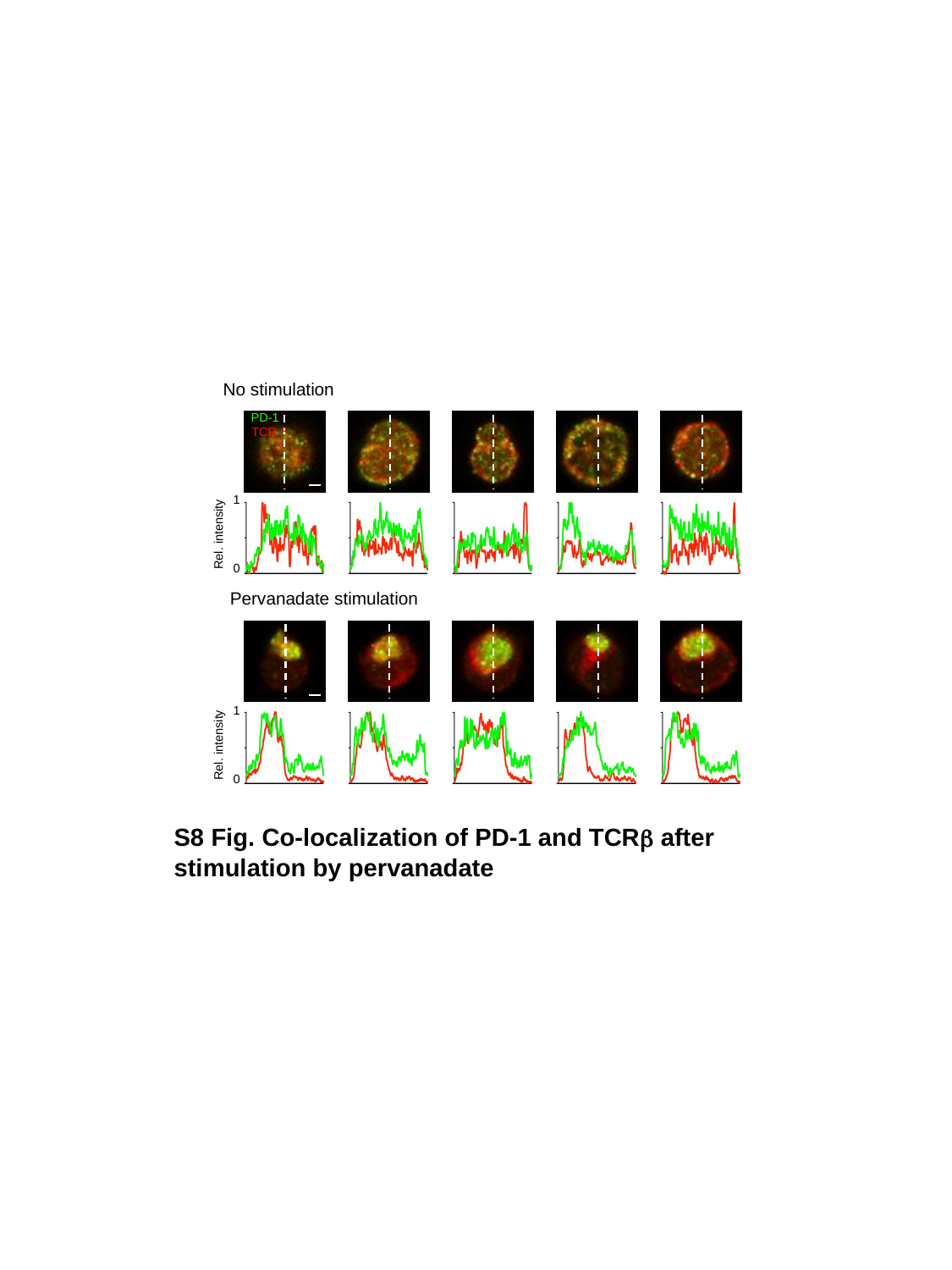

No stimulation
PD-1
TCR β
1
Rel. intensity
0
Pervanadate stimulation
1
Rel. intensity
0
S8 Fig. Co-localization of PD-1 and TCRb after stimulation by pervanadate
