# Supplementary material for: HTLV-1 bZIP Factor Enhances T-Cell Proliferation by Impeding the Suppressive Signaling of Co-inhibitory Receptors
Source: PLoS Pathog. 2017 Jan 3;13(1):e1006120. doi: 10.1371/journal.ppat.1006120 (PMC5234849; doi:10.1371/journal.ppat.1006120)
Supplement: S9 Fig — Interaction between HBZ with SHP-2 (A) or Grb2 (B) was analyzed by immunoprecipitation. Vectors expressing Grb2, SHP-2 and HBZ were transfected into 293FT cells (3.5×10^6^ cells, 10-cm dish). After 48 hours, transfected cells were stimulated with H2O2 for 5 min and cell lysates were immunoprecipitated with anti-Flag or anti-HA antibodies or normal rat IgG as a control. (PPTX) [file ppat.1006120.s009.pptx]

## Slide 1
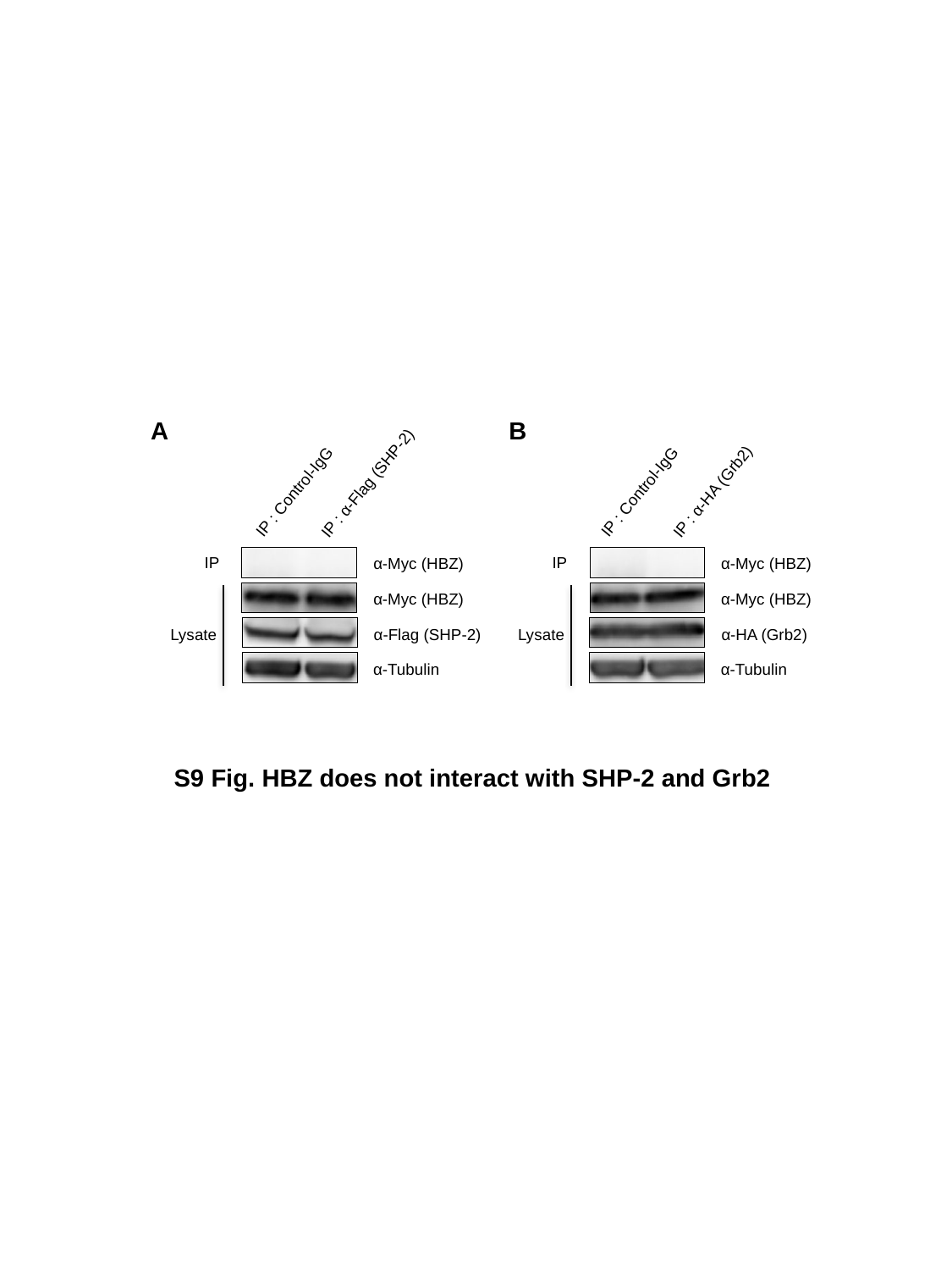

A
B
IP : α-Flag (SHP-2)
IP : α-HA (Grb2)
IP : Control-IgG
IP : Control-IgG
IP
IP
α-Myc (HBZ)
α-Myc (HBZ)
α-Myc (HBZ)
α-Myc (HBZ)
Lysate
Lysate
α-Flag (SHP-2)
α-HA (Grb2)
α-Tubulin
α-Tubulin
S9 Fig. HBZ does not interact with SHP-2 and Grb2
