# Supplementary material for: HTLV-1 bZIP Factor Enhances T-Cell Proliferation by Impeding the Suppressive Signaling of Co-inhibitory Receptors
Source: PLoS Pathog. 2017 Jan 3;13(1):e1006120. doi: 10.1371/journal.ppat.1006120 (PMC5234849; doi:10.1371/journal.ppat.1006120)
Supplement: S10 Fig — (A) THEMIS expression was measured in control Jurkat cells and THEMIS knockdown Jurkat cells by Western blot method. (B) The shRNA-expressing Jurkat cells were seeded into 96-well plates (1×10^4^ cells/well). Cell numbers of each shRNA-expressing Jurkat cells were counted in triplicate by Trypan blue dye exclusion method. (PPTX) [file ppat.1006120.s010.pptx]

## Slide 1
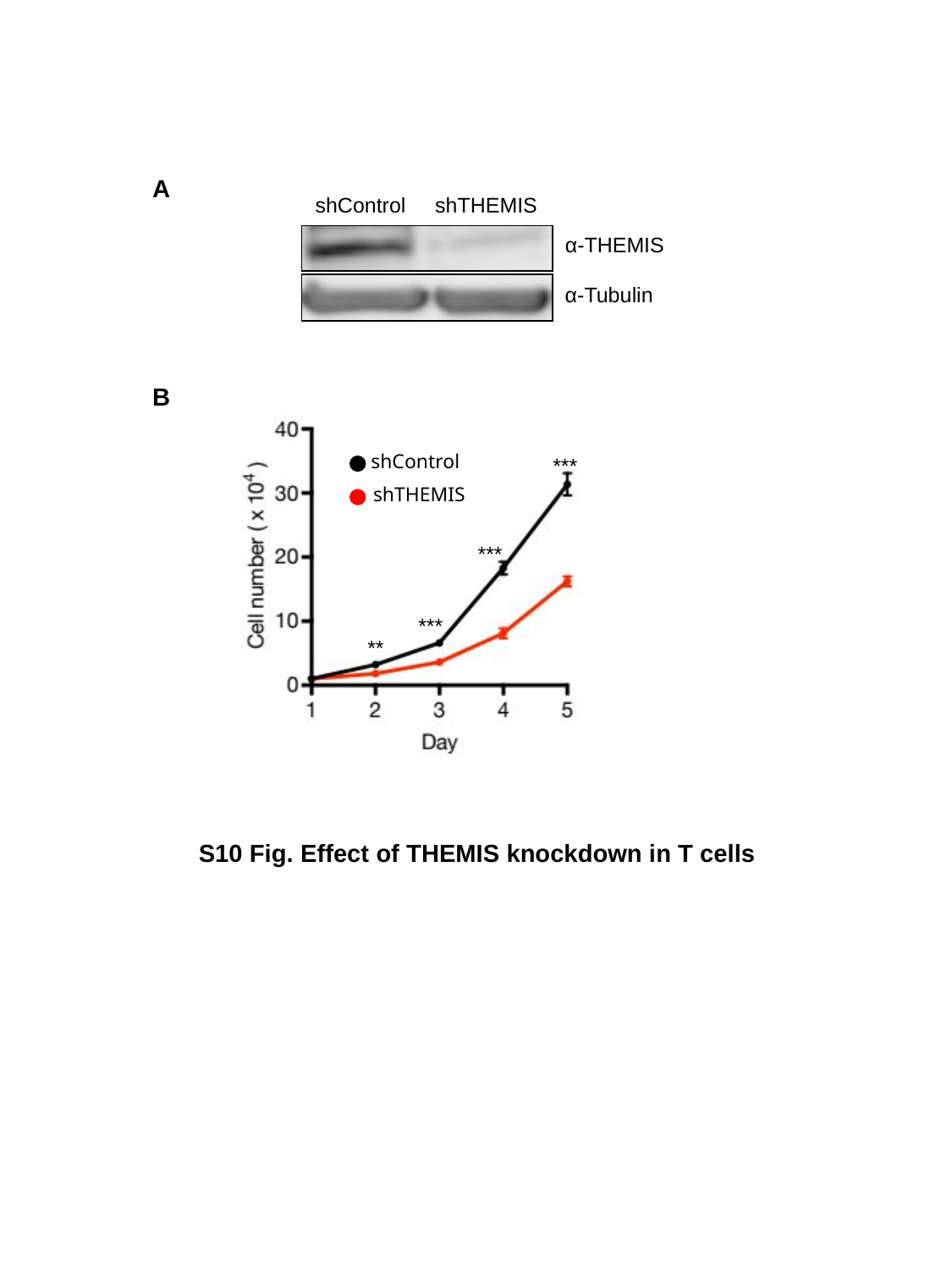

A
shControl
shTHEMIS
α-THEMIS
α-Tubulin
B
shControl
***
shTHEMIS
***
***
**
S10 Fig. Effect of THEMIS knockdown in T cells
