# Supplementary material for: HTLV-1 bZIP Factor Enhances T-Cell Proliferation by Impeding the Suppressive Signaling of Co-inhibitory Receptors
Source: PLoS Pathog. 2017 Jan 3;13(1):e1006120. doi: 10.1371/journal.ppat.1006120 (PMC5234849; doi:10.1371/journal.ppat.1006120)
Supplement: S1 Table — Primers and shRNA target sequences used in this study are shown. (DOCX) [file ppat.1006120.s011.docx]

**S1 Table. Oligonucleotide sequences**

Primers for real-time PCR (human)

|  | Forward | Reverse |
| --- | --- | --- |
| *TIGIT* | CAGGTTCCAGATTCCATTGCTT | ACGATGACTGCTGTGCAGATG |
| *PD-1* | GGATTTCCAGTGGCGAGAGA | GAAAGACAATGGTGGCATACTCC |
| *BTLA* | GAAGAAAACAAACCAGGCATTGT | TTACATTTCTTGCCAGTCTTGAGTTC |
| *LAIR-1* | CACAGTCAATGGACTTCCTGAGAA | ATACGTCACCTCCTGGGAACTC |
| *CD28* | GCGGTCAACCTTAGCTGGAA | CACCCAAAAGGGCTTAGAAGG |
| *ICOS* | GCCTTTGTTGTAGTCTGCATTTTG | GTCTAGATTTTTTGGCTGTGTTCACT |
| *OX40* | AACGACGTGGTCAGCTCCAA | GCCGTGCACAGCTGCTT |
| *r18S* | CGGCGTTATTCCCATGAC | GTGCCCTTCCGTCAATTC |

Primers for real-time PCR (mouse)

|  | Forward | Reverse |
| --- | --- | --- |
| *Tigit* | TCCTGGTGGGATTTACAAGG | AAGCAAATGAGTCCCAGCAC |
| *Pd-1* | CCGCCTTCTGTAATGGTTTGA | GGGCAGCTGTATGATCTGGAA |
| *Btla* | GTGATAATGGGTCGTATAGCTGTTC | GGTCCTGAGGCATTGGTGGCATC |
| *Lair-1* | GTGATTTTCCTCCTTTGTCTTTCC | GCCTCTGCTGCTGTCTTTTGT |
| *Cd28* | TCGGGAATGGGAATTTTACCT | CCCCGTCGCAGTTGAACT |
| *Icos* | CAAGAAAGGAACCTTAGTGGAGGAT | ACGGGTAGCCAGAGCTTCAG |
| *Ox40* | GAGACCCAGCGCCCTACA | AGAAGTCCTGGGCCAGACTGT |
| *β-actin* | CTACAATGAGCTGCGTGTGGCCCCTGA | GAGAGCATAGCCCTCGTAGATGGGCAC |

Primers for RT-PCR (mouse)

|  | Forward | Reverse |
| --- | --- | --- |
| *tax* | ATGGCCCACTTCCCAGGGTT | CCGAACATAGTCCCCCAGAG |
| *HBZ* | ATGGCGGCCTCAGG | GCTTTCTCCCCTGGAGGGCC |
| *β-actin* | CTACAATGAGCTGCGTGTGGCCCCTGA | GAGAGCATAGCCCTCGTAGATGGGCAC |

shRNA sequences

| THEMIS | GAGATCACTGAAGAGCAATAT-CTCGAG-ATATTGCTCTTCAGTGATCTC-TTTTT |
| --- | --- |
| luciferase (control) | CGCTGAGTACTTCGAAATGTC-CTCGAG-GACATTTCGAAGTACTCAGCG-TTTTT |
